# Comprehensive assessment of differential ChIP-seq tools guides optimal algorithm selection

**DOI:** 10.1186/s13059-022-02686-y

**Published:** 2022-05-24

**Authors:** Thomas Eder, Florian Grebien

**Affiliations:** Institute for Medical Biochemistry, University of Veterinary Medicine Vienna, Vienna, Austria

**Keywords:** Differential ChIP-seq, Bioinformatic analysis, Benchmarking differential ChIP-seq tools, Guidelines for differential ChIP-seq

## Abstract

**Background:**

The analysis of chromatin binding patterns of proteins in different biological states is a main application of chromatin immunoprecipitation followed by sequencing (ChIP-seq). A large number of algorithms and computational tools for quantitative comparison of ChIP-seq datasets exist, but their performance is strongly dependent on the parameters of the biological system under investigation. Thus, a systematic assessment of available computational tools for differential ChIP-seq analysis is required to guide the optimal selection of analysis tools based on the present biological scenario.

**Results:**

We created standardized reference datasets by in silico simulation and sub-sampling of genuine ChIP-seq data to represent different biological scenarios and binding profiles. Using these data, we evaluated the performance of 33 computational tools and approaches for differential ChIP-seq analysis. Tool performance was strongly dependent on peak size and shape as well as on the scenario of biological regulation.

**Conclusions:**

Our analysis provides unbiased guidelines for the optimized choice of software tools in differential ChIP-seq analysis.

**Supplementary Information:**

The online version contains supplementary material available at 10.1186/s13059-022-02686-y.

## Background

Protein-DNA interactions are essential for the regulation of most vital processes in living cells. Chromatin immunoprecipitation followed by sequencing (ChIP-seq) [1, 2] is the main tool to identify these interactions on a genome-wide scale. ChIP-seq has been widely used in the life sciences and has led to unprecedented discoveries in development [3, 4], differentiation [3, 5–7], and regeneration [8, 9] as well as in diseases like cancer [10–12] or immune dysregulation [13–15].

The experimental design and the bioinformatic analysis of a ChIP-seq experiment can be focused either on the identification of genomic regions of protein-DNA interactions or on the analysis of their differential occupancy between biological states in a comparative fashion. The latter type of analysis is required for most experimental setups that compare different biological scenarios, such as genotypes, cell states, or treatments. A wide variety of computational tools can be applied for differential analysis of ChIP-seq experiments. While some of these tools were specifically developed for differential ChIP-seq (DCS) analysis, others were initially designed to analyze differential gene expression in RNA-seq datasets, which requires their adaptation for the investigation of ChIP-seq datasets. In principle, all computational tools for DCS analysis are based on certain assumptions. For instance, some tools, particularly those initially developed for RNA-seq analysis, assume that the majority of occupied genomic regions do not differ between experimental states. Yet, this assumption does not hold for comparative ChIP-seq studies that involve experimental perturbation of levels and/or the activity of the protein under investigation. For example, the global downregulation of a particular histone modification after treatment with small molecule inhibitors will result in inadequate identification of differentially occupied regions if the normalization method is based on suboptimal assumptions [16, 17]. Hence, depending on the biological scenario, different normalization methods employed by individual tools will have a strong impact on the outcome. Finally, the length of the identified genomic regions is dependent on the investigated protein, ranging from a few hundred to several thousands of base pairs (bp). All these aspects prevent straightforward decisions to choose the optimal computational tool for DCS analysis for a given scenario. Yet, suboptimal tool usage can have a strong impact on downstream analyses, such as peak annotation and motif analysis. Thus, the guided choice of DCS analysis tools depending on the experiments characteristics is expected to significantly improve the interpretation of ChIP-seq datasets in a biological context.

We set out to comprehensively evaluate the performance of 33 available DCS software tools and custom approaches. Reference datasets obtained by in silico simulation of ChIP-seq tracks were complemented with sub-sampling of experimentally derived ChIP-seq data to obtain realistic representations of background noise in biological data. Tool performances were evaluated with precision-recall curves, and the accuracy of tested tools was assessed depending on peak shape and biological regulation scenario. By combining the area under the precision-recall curve (AUPRC), stability metrics, and computational cost, we derived the DCS score, which enables researchers to choose the optimal DCS analysis tool for any given protein of interest and biological scenario. In addition, our decision trees provide recommendations for the analysis of ChIP-seq datasets of experiments for which no clear assumptions about genomic binding patterns can be made. Thus, our results will lead to improvements in the identification of molecular mechanisms that are based on protein-DNA interactions.

## Results

### Establishing reference data and application of DCS tools

**Fig. 1:**
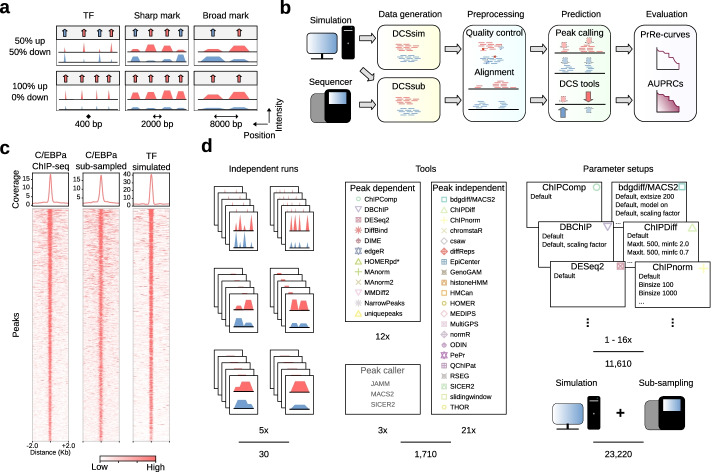
Simulation and sub-sampling of differential ChIP-seq experiments. **a** Schematic overview of simulated peaks and regulation scenarios: Each box represents one test scenario, per scenario the compared samples, and their signal strength are shown in blue and in red. The columns show transcription factor (TF), H3K27ac (sharp mark) and H3K36me3 (broad mark) histone mark ChIP-seq signals (DCSsim width parameters shown below). In the 50:50 regulation scenario, the number of differential regions is equally distributed, while in the 100:0 scenario we assume a global downregulation of the signal. Arrow positions indicate the differential ChIP-seq signals and their color show the sample with the higher signal. **b** Overview of the benchmarking workflow. We applied DCSsim to simulate in silico data and DCSsub to sub-sample genuine ChIP-seq signals. This resulted in sequence reads for two samples (red and blue). After preprocessing, we directly applied peak-independent tools or peak-dependent DCS tools subsequent to peak calling. The resulting peaks and differential regions are depicted as arrows. To assess the DCS tools, we calculated the area under the precision-recall curves. **c** Heatmaps and profile plots showing all peak regions of a ChIP-seq experiment for the TF C/EBPa (left), DCSsub sub-sampling from the same dataset (middle), and the DCSsim simulation of TF peak shapes (right). **d** Quantitative overview of test cases. We generated five independent datasets per peak-regulation scenario. Then we applied three peak callers in combination with 12 peak-dependent DCS tools and 21 peak-independent DCS tools. We used up to 16 parameter setups per DCS tool, and analyses were run for simulated and sub-sampled ChIP-seq data. * HOMER with previously called peaks (HOMERpd)

To model the most relevant biological scenarios, we focused on three common shapes of ChIP-seq signals, representing transcription factors (TF) and two types of posttranslational histone modifications, as proposed by the Roadmap Epigenomics Consortium [18]. While TFs usually occupy genomic regions of a few hundred bp or less [19], histone marks with “sharp” peaks, such as histone H3 lysine 27 acetylation (H3K27ac), H3 lysine 9 acetylation (H3K9ac), or H3 lysine 4 trimethylation (H3K4me3), represent regions covering up to a few kilobases [19, 20]. In contrast, “broad” histone marks, such as H3 lysine 27 trimethylation (H3K27me3), H3 lysine 36 trimethylation (H3K36me3), or H3 lysine 79 dimethylation (H3K79me2) can spread over larger genomic regions of several hundred kilobases [19, 20]. We also defined two biological scenarios that represent frequent experimental conditions in ChIP-seq experiments. First, we assumed that equal fractions of genomic regions show increasing and decreasing signals between two samples (at a 50:50 ratio) while the intensity of remaining peaks does not change. This scenario is representative of comparisons of developmental or physiological states between cells or tissues. In the second regulation scenario, we assumed a global decrease of ChIP-seq peak signals in one sample (in a 100:0 ratio), as is often seen upon gene knockout or pharmacological inhibition of the target protein (Fig. 1a).

We simulated in silico ChIP-seq data for the resulting six scenarios with DCSsim, a Python-based tool we developed to create artificial ChIP-seq reads (Additional file 1: Fig. S1a). Peaks were distributed into two samples representing the biological scenario based on beta distributions and a predefined number of replicates (Additional file 1: Fig. S1b).

To benchmark different DCS tools, we applied DCSsim on the reference sequence of mouse chromosome 19 to simulate 1000 ChIP-seq peaks in two samples and two replicates each. To model a dataset that features more realistic signal-to-noise ratios, a more heterogeneous distribution of background noise, and less clear signal boundaries, we also sub-sampled the top ~1000 ChIP-seq peak regions from genuine ChIP-seq experiments with DCSsub (Fig. 1b). DCSsub is able to sub-sample reads for a defined set of regions (Additional file 1: Fig. S1c), applying the same parameters for the distribution of reads to samples and replicates as DCSsim (Additional file 1: Fig. S1b, d). To model TF peak shapes, we chose ChIP-seq data for the transcription factor C/EBPa [21] (Fig. 1c). The TF CCAAT-enhancer-binding protein alpha (C/EBPa) is involved in myeloid differentiation of hematopoietic stem and progenitor cells [22, 23]. ChIP-seq data for the histone marks H3K27ac [24] and H3K36me3 [25] were chosen to represent sharp and broad marks, respectively (Additional file 1: Fig. S1e, f, g). H3K27ac marks active enhancers and promoters [26] while H3K36me3 is associated with actively transcribed genes [27].

In silico generated and sub-sampled ChIP-seq data (Additional file 2: Table S1) for all scenarios were processed with our evaluation pipeline (Fig. 1b), which includes alignment against the respective reference genomes and peak prediction.

Computational tools for the analysis of DCS data can be divided into two groups depending on their ability to perform peak calling. While peak-dependent tools require previous peak calling through another application, peak-independent tools handle the peak calling procedure internally. We used MACS2 [28], SICER2 [29], and JAMM [30] for external peak calling to match the requirements for detecting different peak shapes from multiple replicates per sample. We then applied 31 available DCS tools plus 2 custom approaches (uniquepeaks and slidingwindow) to the datasets (Fig. 1d, Additional file 3: Table S2, Additional file 4: Table S3). To evaluate the outcomes of DCS tools, we calculated the precision-recall curve per tool and parameter setup. We used the AUPRC (Additional file 1: Fig. S1h) as the main measure of performance. As the exploration of the complete parameter space for each tool and scenario would have exceeded the scope of this work, we chose default and/or recommended parameters and/or adapted them to match respective peak shapes (Additional file 4: Table S3), resulting in 23,220 AUPRC values (Fig. 1d).

### DCS tool performances depend on peak shape and regulation scenario

**Fig. 2:**
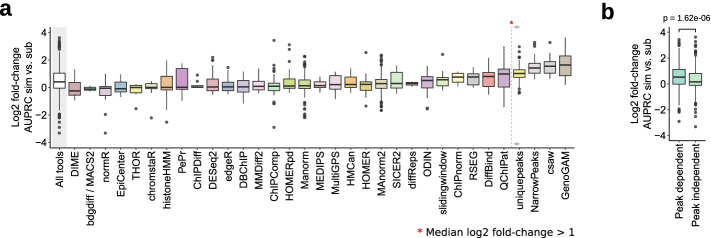
Performance of simulated and sub-sampled input data. **a** Log2-fold change of AUPRC values obtained from DCSsim simulated and DCSsub sub-sampled data. Values >0 indicate a better performance based on AUPRC for simulated data and <0 indicates a higher AUPRC for sub-sampled input. The overall difference for all DCS tools is shown on the left (bar with gray background). Tools were ordered by their median log2-fold change. **b** Comparison of AUPRC values of simulated and sub-sampled data for peak-dependent (*n* = 431) vs. peak-independent (*n* = 1363) DCS tools. *P*-value two-sided Wilcoxon rank sum test. Box plot limits, 25% and 75% quantiles; center line, median; whiskers, 1.5× interquartile range

First, we compared the AUPRCs of all tools and parameter setups derived from simulated and sub-sampled datasets. As expected, most tools performed slightly better when in silico simulated ChIP-seq data were used as input, as peak regions were clearly defined and signal-to-noise ratios were high (Fig. 2a). This was particularly apparent for GenoGAM [31], csaw [32], NarrowPeaks [33], and uniquepeaks. Uniquepeaks is a simple custom approach marking only those regions as differential that were called in one sample and not in the other. However, the performance of several tools was equal when simulated or sub-sampled data were used as input. Next, we focused on the DCS tools with the best performance for each test scenario to investigate specific differences. While simulated data yielded higher AUPRCs for TFs, the results were more heterogeneous when sharp and broad marks were compared (Additional file 1: Fig. S2a). Overall, we found that the difference in performance on simulated vs. sub-sampled regions was significantly higher for peak-dependent tools compared to tools with internal peak calling (Fig. 2b). However, we found that original peak shapes, signal-to-noise metrics, and background uniformity were preserved in sub-sampled data. DCSim also accurately mimicked these parameters (Fig. 1c, Additional file 1: Figs. S1e, f, g, and S2b). Therefore, we decided to combine simulated and sub-sampled data to obtain one performance measure per tool and parameter setup for all subsequent evaluation steps.

**Fig. 3:**
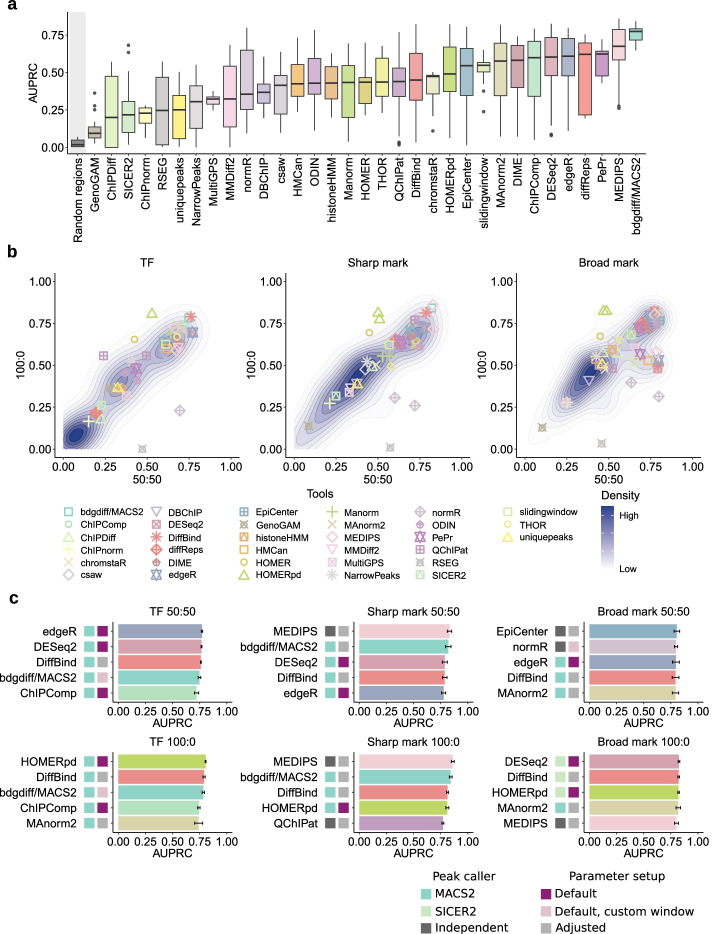
Performance of benchmarked DCS tools based on AUPRC values. **a** Overview of AUPRC values per DCS tool for all test scenarios compared to random regions (see “Methods”), ranked by median AUPRC. Box plot limits, 25% and 75% quantiles; center line, median; whiskers, 1.5× interquartile range. **b** Density plots of AUPRC values per scenario for TFs (left), sharp (middle), and broad marks (right). The two (one for each regulation scenario) top-performing parameter sets per DCS tool are highlighted as colored symbols, remaining data points are visualized as density clouds. **c** AUPRC values of the top five DCS tools parameter sets per scenario (TF left, sharp mark middle, and broad mark right column; 50:50 regulation scenario top and 100:0 bottom row). Colored boxes indicate peak caller, if applicable and if default or adjusted parameter setups were used; whiskers, standard error of the mean

Using this approach, we found that bdgdiff from MACS2, MEDIPS [34], and PePr [35] showed the highest median performance independent of peak shape or regulation scenario (Fig. 3a). Yet, specific parameter setup combinations in several tools yielded superior performance for particular scenarios (Additional file 1: Fig. S3a, b). For example, bdgdiff/MACS2 was outperformed by EpiCenter [36] in identifying broad marks with a 50:50 regulation scenario or by edgeR [37] in finding differential TF peaks with a 50:50 regulation scenario (Additional file 1: Fig. S3b). To obtain more detailed insights into the performance of DCS analysis tools under specific conditions, we first focused on their performance depending on the peak shape. Highlighting the optimal parameter setup for each tool in a density plot of all AUPRC values revealed a linear relationship between AUPRC values in the 50:50 and 100:0 scenarios for both TFs and sharp marks, with the exception of a few outliers (Fig. 3b, left and middle panels). In contrast, comparison of AUPRC values from datasets with broad marks showed that a substantial number of tools performed better in the 50:50 regulation scenario (Fig. 3b, right).

DiffBind [38], bdgdiff/MACS2, edgeR, or DESeq2 [39] were prominently represented among the top five tool parameter setup combinations based on AUPRC values across all conditions (Fig. 3c, top tools with exact parameter setups are shown in Additional file 1: Fig. S3b). HOMERpd [40] (peak-dependent run) and DiffBind, both tools with previous peak calling via MACS2 or SICER2, yielded very good results for the 100:0 scenarios, irrespective of peak shape. Finally, MEDIPS performed very well in the analysis of sharp marks.

**Fig. 4:**
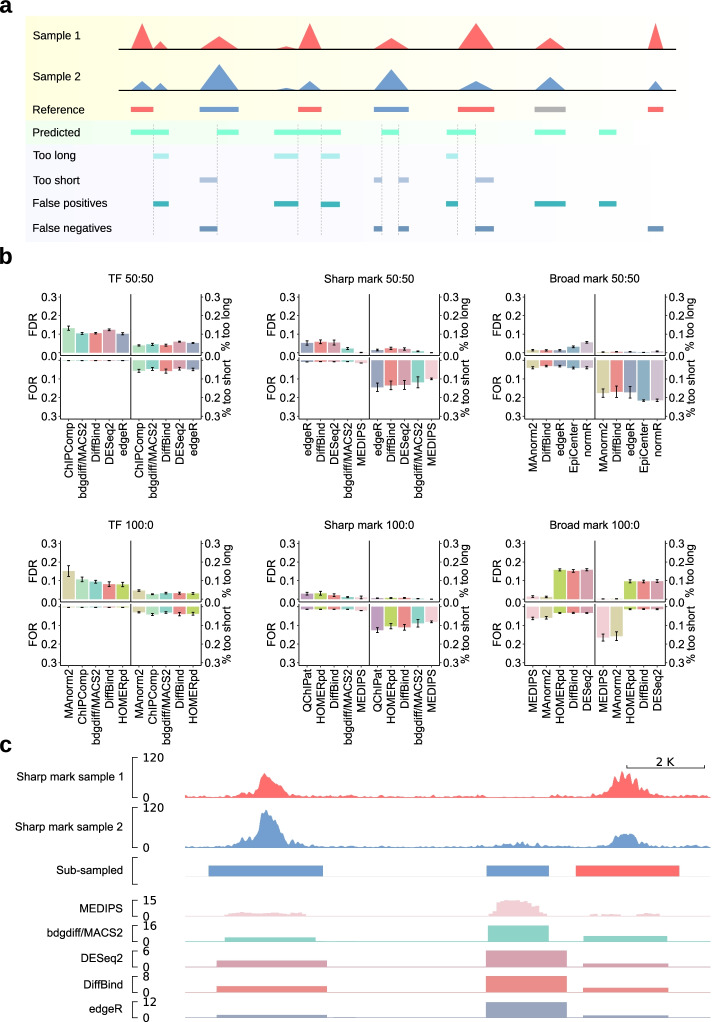
Accuracy profiles of DCS tools. **a** Schematic representation of accuracy profiles. Rows with yellow background show simulated or sub-sampled ChIP-seq signals. The reference region highlights the sample color with the higher signal. Regions with no difference are depicted in gray. The predicted regions from a DCS tool are highlighted with green and the calculated accuracy metrics with blue background. We investigated false positives, false negatives, and too long and too short regions, representing the false positive and false negative base pairs (bp), respectively with the constraint that the predicted regions overlapped with a reference region. **b** Bar charts show the false discovery rate (FDR), the false omission rate (FOR), the percentage of too short, and the percentage of too long bp for the best-performing parameter sets of the top 5 DCS tool parameter combinations per scenario (from left/5th to the right/1st) based on AUPRC. TFs (left), sharp marks (middle), and broad marks (right) in the columns and 50:50 regulation (top) as well as 100:0 regulation (bottom) in the rows. Whiskers represent the standard deviation. **c** Example coverage plot of DCSsub sub-sampled H3K27ac reads (samples in row 1 (red) and 2 (blue)) representing sharp marks with the respective reference regions (row 3). In row 3 upregulation in sample 1 is indicated in red, downregulation in blue. Rows 4 to 8 show predicted regions from the best parameter setups of the top 5 DCS tools for sharp mark data and 50:50 regulation. The height of predicted regions represents the − log10 of *p*-value, adjusted *p*-value, or FDR or the score derived from the respective DCS tool. Higher bars represent higher confidence in the indicated region

To extend the results obtained from the AUPRC analyses, we interrogated how well the predicted differential regions matched the reference. As DCS tools rank their results based on various metrics, such as *p*-value, FDR, or diverse scores, it is not possible to rank predicted regions using a uniform threshold. Therefore, we focused on the top 300 predicted regions, as at least 300 differential regions were classified by the simulation or sub-sampling procedures in all test cases. We calculated the false discovery rate (FDR) as the fraction of false positive bp and the false omission rate (FOR) as the fraction of false negative bp in individual peaks (Fig. 4a). To measure how precise individual DCS tools predicted reference regions, we calculated the percentages of predicted bp that are shorter or longer than the respective reference region in significantly different regions (Fig. 4a). As peak-dependent tools can only work with the input regions they receive from peak calling algorithms, several combinations did not yield meaningful results. For example, differential TF peaks could not be accurately predicted with edgeR subsequent to peak calling with SICER2 (Additional file 1: Fig. S4a) as this tool was designed for calling broad peaks. Furthermore, as a subset of DCS tools depends on a predefined window size, this affects their predictions. For instance, when EpiCenter was executed with broad mark data, all parameter setups were tested. A very narrow window size of 100 (parameter setups 1 and 3) resulted in a high number of predicted regions and only the full dynamical expansion window parameter (parameter setup 2) predicted regions that closely resembled the sub-sampled reference (Additional file 1: Fig. S4b). The FDR was below 0.2 in all cases and even lower for most histone marks, while the FOR was lower than the FDR across most samples (Fig. 4b). This was caused by the relatively large part of the chromosome that was devoid of ChIP-seq signals. The FOR increased with broader peak shapes. The fraction of too short predictions was increased for sharp and broad histone marks, while the percentages of too long or too short predictions were balanced for TF peaks (Fig. 4b). Broad marks at a 100:0 regulation scenario represented an exception: here we found that HOMERpd, DiffBind, and DESeq2 yielded higher FDR values and an increase in too long predictions. In contrast, MEDIPS and MAnorm2 [41] generated higher FOR values and higher percentages of too short predictions. This is likely caused by different peak calling strategies leading to the best AUPRC results in this test set. In the top-ranking DCS tool-peak caller combinations in this scenario, SICER2-derived peaks were used for the analyses with HOMERpd, DiffBind, and DESeq2, and MACS2 peaks were used for MAnorm2, while MEDIPS is a peak-independent tool. Hence, in this scenario, peak calling with SICER2 on broad mark data increased the fraction of false positives and too long predictions but decreased the fraction of false negatives and too short predictions for the top DCS tool setups. Thus, these data show that inaccuracies in the prediction of genomic regions are asymmetrically distributed depending on the DCS tool and peak caller (Fig. 4c, Additional file 1: Fig. S4c, d).

### Higher AUPRC positively correlates with higher signal-to-noise ratio

**Fig. 5:**
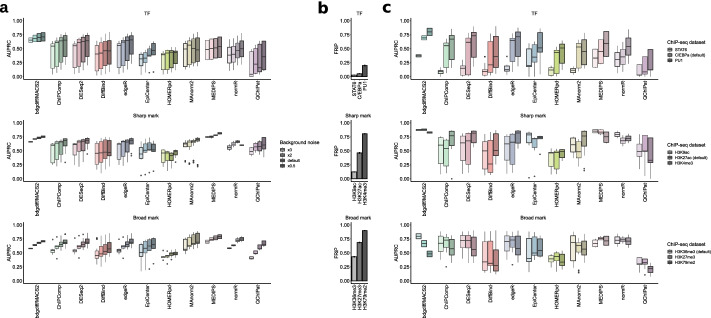
Influence of FRiP on the performance of DCS tools. **a** AUPRCs of the top 11 DCS tools (based on AUPRC for the initial six shape and regulation scenarios) depend on the background noise. Boxplots per DCS tool are ordered by noise level, from high to low. **b** FRiP in the sub-sampled datasets. Darker color represents higher FRiP. Top panel, TFs; middle panel, sharp marks; bottom panel, broad marks. Whiskers indicate the standard deviation. **c** AUPRCs for sub-sampled ChIP-seq data from different TFs and histone marks depend on FRiP. Boxplots per DCS tool are ordered by FRiP from low to high. Top panels, TFs; middle panels, sharp marks; bottom panels, broad marks. Box plot limits, 25% and 75% quantiles; center line, median; whiskers, 1.5× interquartile range

Another important aspect in DCS analysis is the signal-to-noise ratio of the investigated data. This parameter is typically assessed by the fraction of reads in peaks (FRiP) [42] which represents the ratio of mapped reads within called peaks to all usable reads in the sample. To test how the benchmarked DCS tools handle datasets with different signal-to-noise ratios, we simulated ChIP-seq data with a 50:50 regulation scenario. We created datasets with TF, sharp, and broad mark peak shapes with half, two, and three times the level of background signal (i.e., the noise) of the original test sets. For the evaluation, we chose the top 5 DCS tools for the original six test scenarios (Fig. 3c). As expected, decreasing the background noise resulted in a homogeneous increase of performance for the majority of DCS tools (Fig. 5a, Additional file 1: Fig. S5a). However, there were some exceptions: The AUPRCs for HOMERpd were largely unaffected by the level of background noise. Normr, EpiCenter, and DiffBind exhibited lower AUPRCs associated with low noise when sharp marks were analyzed, and no consistent pattern of correlation between median AUPRC and noise level was observed for MAnorm2 and QChIPat in the analysis of TF peaks (Additional file 1: Fig. S5a).

While these experiments allowed us to precisely control the global signal-to-noise ratio, the background noise is often unequally distributed in real datasets. To investigate the effect of this on DCS analysis, we analyzed additional sets of real ChIP-seq data with different signal-to-noise ratios. For TF data, we included ChIP-seq results for STAT6 and PU1 [43], which are factors involved in the differentiation of hematopoietic cell types. For additional sharp marks, we chose ChIP-seq data for H3 lysine 9 acetylation (H3K9ac) (Wang Y, Sun Z: Gestational choline supplementation improves cross-generational mood by epigenetic upregulation of Nr3c1, unpublished) and H3 lysine 4 trimethylation (H3K4me3) [43], which are histone modifications associated with transcriptional activation. As broad marks, we selected ChIP-seq data for H3 lysine 27 trimethylation (H3K27me3) [44], which is a marker for gene repression and H3 lysine 79 dimethylation (H3K79me2) [45], which is closely correlated with transcriptional elongation. As expected, AUPRCs of most DCS tools increased with higher FRiP for TF datasets (Fig. 5b, c, Additional file 1: Fig. S5b). The only exception was MAnorm2, where the median AUPRC was decreased for the dataset with the highest FRiP. Although the differences in FRiP were larger in sharp mark data than in the TF sets, no uniform correlation of AUPRC values with higher FRiPs was observed (Fig. 5b, c, Additional file 1: Fig. S5b). For broad histone marks, we noticed stable or even decreased AUPRCs for datasets with higher FRiP (Fig. 5b, c, Additional file 1: Fig. S5b). Notably bdgdiff from MACS2 received lower AUPRC for the sub-sampled broad mark samples with higher FRiP. In summary, these experiments show that increasing FRiP is not always positively correlated with the performance of DCS tools.

### Chromosome characteristics influence the performance of DCS tools

Another important aspect in DCS analysis is the characteristics of ChIP-seq signals deriving from chromosomes that differ in size and nucleotide composition. To test the influence of these parameters, we simulated peak sets for TFs, sharp and broad marks with a 50:50 regulation from four additional mouse chromosomes. As our initial analyses were based on chromosome 19 (61.431.566 bp, 43% GC), which represents the shortest mouse chromosome, we added the longest chromosome (chr1: 195.471.971 bp, 41% GC). We also added three chromosomes with divergent size and GC content (chrX: 171.031.299 bp, 39% GC; chr11: 122.082.543 bp, 44% GC; chr8: 129.401.213 bp, 42% GC) (Additional file 1: Fig. S6a). Results for the top DCS tools of the initial analysis showed that AUPRCs were relatively stable, indicating that DCS tool performance is largely unaffected by diverging chromosome parameters (Additional file 1: Fig. S6b). Notably, analysis of chromosome X with several tools yielded slightly decreased AUPRC values for TFs, sharp and broad marks.

**Fig. 6:**
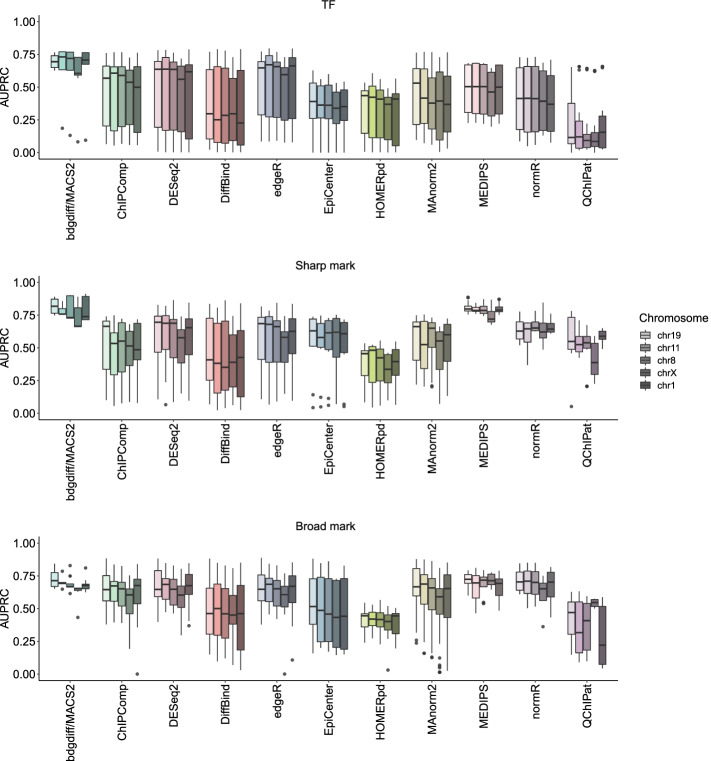
Chromosome characteristics and signal distribution influence AUPRC. Combined AUPRCs from simulated and sub-sampled data of the top 11 DCS tools (based on AUPRC of the initial six shape and regulation scenarios) for five chromosomes of mm10 (chr1, chr8, chr11, chr19, and chrX) for TFs, sharp, and broad marks are shown. Chromosomes per DCS tool are ordered by length, from short to long. Top panel, TFs; middle panel, sharp marks; bottom panel, broad marks. Box plot limits, 25% and 75% quantiles; center line, median; whiskers, 1.5× interquartile range

To further investigate the uniformity of ChIP-seq signals between chromosomes, we also sub-sampled ChIP-seq data for C/EBPa (TF), H3K27ac (sharp mark), and H3K36me3 (broad mark) from five representative chromosomes of the mouse genome. Despite a higher degree of heterogeneity, differences in AUPRCs between chromosomes from the sub-sampled data were comparable to the results obtained with simulated data (Additional file 1: Fig. S6c). This included the exceptional behavior of chromosome X. Combination of the results from the simulation and the sub-sampling confirmed that neither size nor GC content strongly influenced the performance of DCS tools (Fig. 6). The slight reduction in AUPRCs for chromosome X was preserved in the majority of tools and QChIPat resulted in the most heterogeneous distribution of AUPRCs. Lastly, no obvious correlation between chromosome length or GC content and the AUPRC of the top-ranking tools could be observed (Additional file 1: Fig. S6d). In summary, these analyses show that chromosome and ChIP-seq signal characteristics affect the AUPRC of the top-ranking tools, while we did not observe any clear advantage of specific tools for particular scenarios and/or settings.

### Different DCS tools show varying computational requirements

**Fig. 7:**
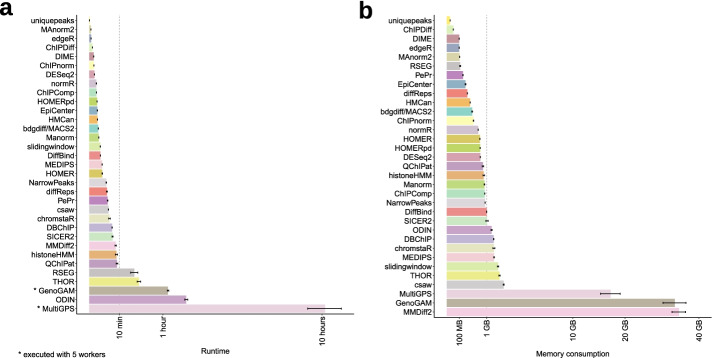
Runtime and memory requirements of benchmarked DCS tools. **a** Average runtime and **b** memory consumption of all benchmarked DCS tools over the six tested scenarios. Due to their extensive runtimes, GenoGAM and MultiGPS were executed with 5 workers. Whiskers indicate standard error of the mean

Next, we assessed the runtime and memory requirements of each computational tool for DCS analysis. Whereas the majority of tools completed the analysis of the initial small test datasets within minutes, three tools required more than 1 h of processing time (Fig. 7a). All tools were run with a single CPU, with the exception of GenoGAM and MultiGPS [46] for which five threads were used to complete the analysis in a reasonable time frame. Furthermore, the time required for preparation and/or reformatting of input files was different for the investigated tools. However, on average, less than a minute was required for this step (Additional file 1: Fig. S7a). The majority of tools required between 100 MB and 2 GB of memory to process the benchmark datasets (Fig. 7b). Only MultiGPS, GenoGAM, and MMDiff2 [47] required significantly more memory, ranging from 17 to 34 GB. As expected, most tools required less memory and completed faster when processing TF peaks in comparison to sharp or broad marks, as the latter comprised 2.0- to 2.4-fold more reads (Additional file 1: Fig. S7b, c).

### Overview of the performance of DCS tools highlights individual strengths and weaknesses

**Fig. 8:**
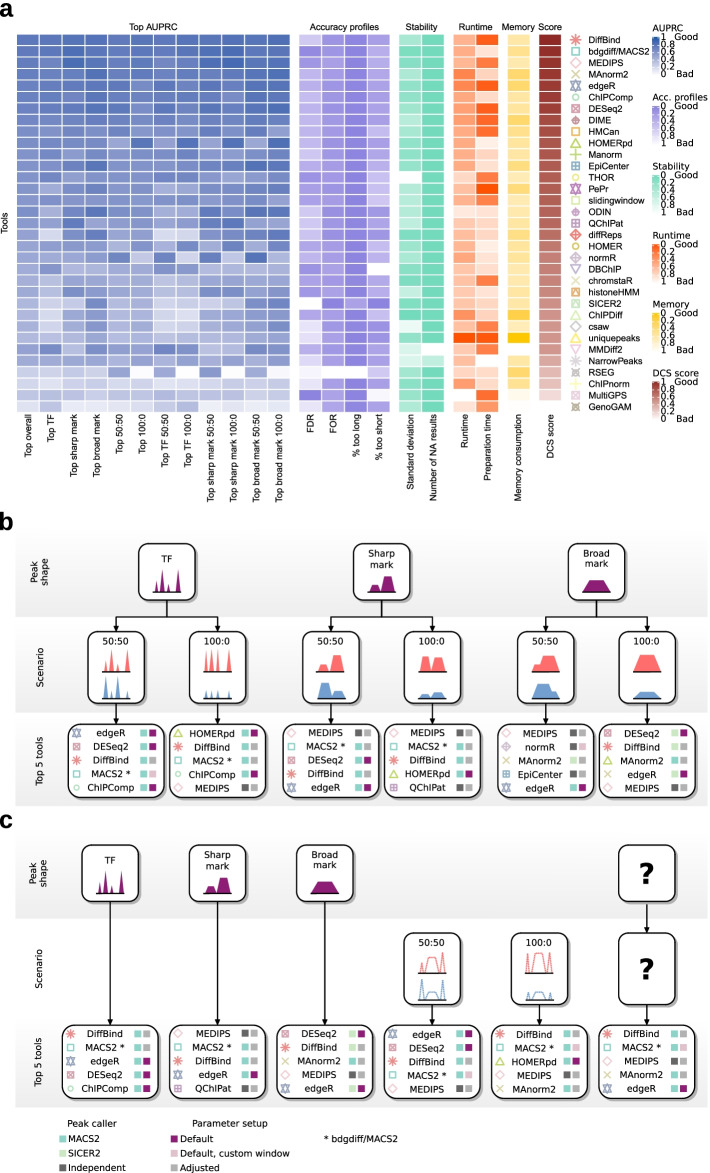
DCS tool performance and guidelines for DCS tool selection. **a** Heatmap summarizing DCS tool performance. Columns represent top AUPRC values, accuracy profiles, stability, runtime, memory consumption, and mean DCS score of the benchmarked DCS tools. The AUPRC of the best parameter setup per DCS tool is shown for peak shape and regulation scenarios and their respective combinations. All other metrics are shown as average of all parameter setups per DCS tool over all test scenarios. Standard deviations were calculated between AUPRCs of the simulated and sub-sampled replicates. The number of NA results summarizes all failed and faulty execution runs or runs with empty outputs. Preparation time represents the time to process the input files preceding DCS prediction. Tools were ordered by their mean DCS score over all test sets. **b**, **c** Decision trees listing top-performing parameter setups per DCS tool based on DCS score to guide investigators towards the five top-ranking DCS tools and their parameter setups depending on peak shape and regulation scenario. **c** Decision tree for situations where shape, regulation, or both are unknown. Here, the ranking is based on DCS score of the combined regulation scenarios for TFs, sharp, and broad marks, the combined peak shapes for 50:50 and 100:0 regulation and over all tested scenarios for situations where shape and regulation are unknown. Colored boxes indicate the applied peak caller for the respective parameter setup and if default, default with custom windows, or adjusted parameters should be used. For detailed information on the setups, see Additional file 4: Table S3 and Additional file 6: Table S5

To obtain a comprehensive overview of the performance of individual DCS tools, we highlighted the AUPRCs for the top parameter setup per tool for each regulation scenario and peak shape (Fig. 8a). We also combined top AUPRC values for peak shapes, regulation scenarios, and over all datasets (Fig. 8a). In addition, we calculated the average performance over all parameter setups per tool (Additional file 1: Fig. S8a). Furthermore, we visualized the accuracy profiles and the stability metrics, which include the standard deviation between the five independent predictions and the number of non-successful runs, as average per tool (Fig. 8a, Additional file 1: Fig. S8b). The computational requirements are represented as average runtime, preparation time, and memory usage. This global analysis revealed that the majority of tools showed a solid performance. The top 10 tools based on the top overall AUPRC values were DiffBind, bdgdiff/MACS2, MEDIPS, MAnorm2, edgeR, ChIPComp [48], DESeq2, DIME [49], HOMERpd (peak-dependent run), and HMCan [50].

MMDiff2, GenoGAM, and DBChIP represent examples of tools that yielded higher AUPRC for datasets with TF peaks in comparison to other peak shapes [47]. Conversely, e.g., ChIPDiff [48] and QChIPat [49] predicted sharp and broad peaks with a better performance than TF peaks. Increased AUPRC values for broad over sharp and TF peaks were observed for SICER2. This was expected, as this tool was initially designed for the analysis of histone mark peaks.

Two patterns emerged when the performance of DCS tools was compared based on the biological scenario. HOMER and HOMERpd (executed with previously called peaks) showed increased AUPRC values in the 100:0 scenarios, while RSEG [51], MAnorm2, and normR [52] yielded better results for the 50:50 regulation scenario. In particular, RSEG was not able to successfully deal with a global downregulation of ChIP-seq signals in one sample.

The FDR values ranged between 0.026 and 0.761 for all investigated tools. Data from SICER2, RSEG, GenoGAM, ChIPnorm, csaw, uniquepeaks, and NarrowPeaks generated FDR values higher than 0.5. FOR rates were between 0.009 and 0.029, with the maximum value of 0.078 represented by RSEG as the only outlier. When focusing on predictions that were too long, RSEG showed increased values. However, this was due to aberrant results from the 100:0 scenario. Without this outlier, the FOR values ranged from 0.0001 to 7.24%. The fraction of predicted regions that were shorter than the reference was between 0.002 and 0.31%. The highest values arose from DBChip and MultiGPS with 0.31% and 0.29%, respectively. The standard deviation of the AUPRCs between replicates was generally low, ranging from 0.008 (ChIPDiff) to 0.061 (csaw). There were no empty output files or failed execution runs for most tools, except for a few failed runs with MMDiff2 (14.6%) and NarrowPeaks (7.6%). MMDiff2 memory allocation failed for individual peak files for broad or sharp histone marks and NarrowPeaks sporadically exited with inconclusive error messages.

### A guide to selecting optimal DCS tools depending on peak shape and regulation scenario

While the AUPRC values represent the performance of a given DCS tool/setup with high precision, the stability metrics, runtime, and memory usage also need to be taken into account for the practical application. Therefore, we introduce a weighted score (Additional file 1: Fig. S8b, for weights see “Methods”) that combines all aforementioned metrics. Assuming that for the practical application of a DCS tool precision and recall are the most important factors, the DCS score prioritizes AUPRC over runtime and memory consumption. Runtime was given more weight than preparation time, number of NAs, and standard deviation (values used for Additional file 1: Fig. S8a in Additional file 5: Table S4 and Additional file 6: Table S5). We utilized the resulting values to guide investigators towards the optimal choice and setup of a DCS tool that is tailored for the type of data under investigation in two decision trees (values and ranking in Additional file 6: Table S5). The first tree shows the top five DCS tool setups for different peak shapes and regulation scenarios (Fig. 8b). In the second tree, we highlight the top five DCS tool setups for the analysis of ChIP-seq datasets for which peak shape and/or regulation is unknown (Fig. 8c).

## Discussion

While several publications have compared small subsets of software tools for DCS analysis [31, 32, 35, 36, 41, 46–48, 50, 52–61], only one study has investigated a larger set of 14 tools at a low level of comprehensiveness [62]. In this work, we set out to benchmark available DCS tools by investigating their predictions on in silico simulated and sub-sampled genuine ChIP-seq data representing the three most prominent peak shapes and regulation scenarios. Using this setup, we provide a comprehensive characterization of 33 DCS tools and approaches through AUPRCs together with information about their stability, runtime, and memory usage. By merging these parameters into a combined DCS score, we provide decision trees that will help to guide biomedical scientists, biologists, and bioinformaticians alike, identifying the optimal tool for comparative analysis of ChIP-seq data.

Based on the final DCS score, we did not identify a single DCS tool that showed superior performance across all regulation scenarios and peak shapes. In addition, we found that the choice of parameter setup among individual DCS tools could have a massive influence on the accuracy of the results. Yet, multiple tools robustly ranked among the best candidates in many conditions tested by us. DCS analysis using previously called peaks showed only a slight advantage over peak-independent tools. Bdgdiff from MACS2 clearly stood out as one of the most robust tools across different scenarios and input parameters. In this case, the quality of the results was mostly dependent on the peak calling setup to match the peak shape of the analyzed data. However, robust peak calling was a prerequisite for optimal performance in differential peak prediction for all tools.

As expected, a decrease in noise led to an increase in AUPRC values in both simulated and sub-sampled data. While this correlation was very high when TF peaks were analyzed, it was less conserved for sharp marks and almost absent upon evaluation of broad mark peak sets. In particular, the performance of MACS2 dropped with increasing FRiP for broad marks. The reason for this might be that MACS2 was designed to work with TFs and sharp marks. Thus, irrespective of the FRiP, it is possible that peaks, subsections of peaks, or summits of particular broad mark ChIP-seq data (e.g., H3K36me3) feature characteristics, which MACS2 cannot process in an efficient fashion. In general, the linkage of AUPRCs to the individual characteristics of histone mark ChIP-seq datasets appeared to be stronger than to the respective FRiP.

Consistent differences in DCS tool performance between chromosomes were only observed when autosomes were compared to chromosome X. The lower AUPRC in ChIP-seq data derived from the X-chromosome might result from higher numbers of unknown bases (number of Ns: 4.41% in chrX vs. 2.99% mean of mm10), scaffolds, and unspanned gaps in its assembly in mm10. In particular, QChIPat yielded very heterogeneous results between chromosomes for different peak shapes. This could be caused by the species parameter included in this tool, which is limited to the whole genomes of mouse or human. However, as in typical DCS analyses all chromosomes of a given species are investigated, this effect might be mitigated in real-life scenarios. In general, however, these results indicate that for optimal tool performance, it is recommended to appropriately adjust the parameters specifying genome length, effective genome size, or species.

To aid investigators in their choice of the most appropriate tool for DCS analysis, we present the most suitable tool combinations for individual peak shapes and scenarios that were simulated and sub-sampled in our study. We combined our assessment criteria to suggest tools that are best suited to analyze ChIP-seq data of unknown peak shape and/or regulation scenario. To investigate ChIP-seq signals with mixed peak shapes, we recommend using the respective top-ranking tools. As correct peak detection is a prerequisite for high AUPRCs, concurrent peak calling with different setups is also an option, as this can be used to match the major peak shape types. For this case, users are advised to stick to the suggested peak-dependent DCS analysis tools in the respective regulation scenario with unknown peak shapes.

Despite their good performance in selected scenarios, tools applying predefined bin- or window-sizes like MEDPIS, normR, QChIPat [60], or EpiCenter predicted multiple short regions per individual broad peak region. Predictions from these tools are linked to the predefined bin size, which can be several orders of magnitude shorter than the actual peaks. While the sum of all short predictions may provide sufficient coverage of the reference region, this situation might lead to ambiguous situations in the downstream analysis of data, e.g., in peak annotation and motif enrichment analysis.

Our findings and recommendations are expected to be transferable to the comparative analysis of NGS technologies that produce coverage signals similar to ChIP-seq, such as DNase-seq [63], Mnase-seq [64], FAIRE-seq [65], DamID-seq [66–68], and CUT&RUN [69]. Also in differential ATAC-seq [70], the type of applied normalization was shown to greatly influence the identification of differentially accessible genomic regions [71]. As ATAC-seq signals often share characteristics of sharp mark ChIP-seq peaks, investigators might choose the tools, which showed best performance in this section, such as MEDIPS, bdgdiff/MACS2, or DiffBind for the analysis of ATAC-seq data. Furthermore, in single-cell ATAC-seq [72, 73] data, it might be appropriate to stick to the guidelines for sharp marks without a fixed regulation scenario. Consequently, our recommendations are also applicable for single-cell ChIP-seq [11, 74] data analysis. As the regulation scenarios could be diverse, we suggest using the tools that show highest accuracy in situations where only the peak shape is known.

## Conclusions

This benchmarking study and our recommendations represent a comprehensive guideline for the choice and setup of the optimal software for DCS analysis that is tailored to the data at hand. Our recommendations cover the most prevalent peak shapes and the most relevant biological scenarios. This work is expected to benefit all investigators applying DCS analysis and will lead to improvements in the identification and characterization of protein-DNA interactions. Hence, this will help in the identification of molecular mechanisms of gene regulation in health and disease.

## Methods

### DCSsim

DCSsim generates in silico ChIP-seq reads for two samples with two or more replicates. It uses a user-defined reference sequence with additional control input reads. Spike-in reads from a user-defined additional species can be added. DCSsim was established by object-orientated programming based on a script created for the THOR manuscript [61] with the following modifications: A modified version of the weight distribution was used for input generation. We added read objects, which was better suited for the object-oriented programming style used by us. We extended the process of how reads were distributed into two samples through the beta distribution and the dispersion into replicates using the Dirichlet distribution. We also added the option to change parameter values, which enabled us to simulate different scenarios and implemented multi-threading support.

Clusters of binding sites in this context are sets of one or more protein-DNA interactions in close proximity in the genome sequence. Clusters were initialized by picking a random position in the provided genome sequence. The number of proteins in one cluster was drawn from a multivariate normal distribution and positions were checked against a blacklist, which can be provided by the user. In the next step, the number of proteins at the defined positions was initialized. Inside a protein object, the number of fragments was drawn from a combination of a lognormal and a gamma distribution and fragments were distributed into two samples via a beta distribution. The number of fragments was scaled based on the results of a Laplace distribution. Alternatively, beta results were scaled based on an exponential distribution. The first option does not touch the (beta) distribution into the two samples, while the latter option does not change the number of fragments. There is also the possibility to apply no scaling. Next, individual fragments were initialized and distributed to sample 1 and sample 2 as well as into the defined number of replicates based on the result of the beta distribution. This was achieved via a Dirichlet distribution, which is the multivariate version of a beta distribution. Every fragment received a random shift in its position, which can be modified via a set of parameters. In addition, background noise was added to the samples and their replicates. This was done via weighted bins, which were also used to create the noise in input objects for both samples. Noise weights were sampled from a gamma distribution. DCSsim is also able to construct spike-in reads from different organisms using a similar approach as is used for generating input samples. Finally, reads were randomly chosen from both ends of the fragments and written to fasta files together with noise and spike-in reads. The positions of peaks as well as their regulation status were stored in bed files, and a general report with tables and histograms was stored in pdf format.

DCSsim supports multi-threading as well as simulating in batches to account for limited memory space on the used machine. A set of parameter simulation scripts that enable distribution sampling without creating reads but creating diverse histograms and plots to save time for parameter estimation can be found at https://github.com/Edert/DCSsim.

### Simulation

DCSsim and DCSsub parameters were chosen to match real ChIP-seq experiments. For each of the DCS scenarios, we performed five simulations creating independent test sets. We applied DCSsim for chromosome 19 of the mouse genome sequence (mm10) with repeat-masked regions greater than 1 kb as a blacklist. We simulated 1000 peaks in two replicates per sample. The final reads had a length of 50 bp. The minimum reads per region was set to 10 reads, and a threshold of 0.7 for being differentially occupied was used. For tools capable of handling scaling factors, we also simulated spike-in reads from *Drosophila melanogaster*.

For TF peaks, fragments were designed to have a mean length of 200 bp with a standard deviation of 50. Fragment count parameters based on the fits of the real ChIP-seq data were --frag-count-sh 2.23281 --frag-count-sc 12.43632 --frag-count-op 0.5 --frag-count-om 3.7 --frag-count-os 0.9. Protein counts per cluster were set to 1, and the protein size for TF peaks was 400 bp. Beta for the 50:50 scenario was set to 0.5|0.5 and 6.0|0.5 for the 100:0 scenario. Scaling was set to “frag” and exponential distribution parameters: --frag-count-ex-loc 10 --frag-count-ex-scale 200. Background noise was set to 0.75 and spike-in was true with the *Drosophila melanogaster* chromosome 6 (dm6_chr2L) and a coverage of 0.25.

For sharp marks, all parameters were similar to those of TF peaks except the fragment count parameters were as follows: --frag-count-sh 1.35985 --frag-count-sc 56.71249 --frag-count-op 0.5 --frag-count-om 7.2 --frag-count-os 0.9 and protein size was 2000 bp. Scaling was set to frag and --frag-count-ex-loc 10 --frag-count-ex-scale 5000. Fragment distances were on and set to --frag-dist-muno-mean=900,900 --frag-dist-muno-cov “50000,0;0,80000”. Background noise was set to 1.5.

Broad mark peaks were also simulated like TF peaks with the difference that the fragment count parameters were as follows: --frag-count-sh 1.72598 --frag-count-sc 210.25902 --frag-count-op 0.5 --frag-count-om 6.6 --frag-count-os 0.9 and protein size was 8000 bp. Scaling was --frag-count-scaling=“frag” --frag-count-ex-loc 10 --frag-count-ex-scale 2500. Fragment distances were switched on and --frag-dist-muno-mean=3000,5000 --frag-dist-muno-cov “1000000,0;0,1000000”. Background noise was set to 1.5.

For the different FRiP scenarios, we used the same parameters as described above and set --back-avg to 0.375, 1.50, and 2.25 for 0.5×, 2×, and 3× background signal respectively for TFs. For sharp marks and broad marks, we set --back-avg to 0.75, 3.0, and 4.5. The four additional chromosomes were also simulated as described above and the -c parameter was set to chr1, chr8, chr11, and chrX depending on the chromosome used for the simulation.

### DCSsub

DCSsub generates DCS reads for two samples in two or more replicates. This is done by non-random sub-sampling data from one genuine ChIP-seq bam file plus one or more ChIP-seq input control bam files. For all described experiments, the ChIP-seq bam file was obtained by merging multiple alignment files (replicates) of the same TF or histone mark (for more details see “Sub-sampling” section) via BEDtools [75] merge. As DCSsub uses a more or less reduced fraction of the original sequence reads from the supplied alignment bam files, we refer to the output data as sub-sampled. Only regions listed in the supplied bed file were processed into differential regions. For each region, the overlapping and aligned reads were included into the sampling pool. The number of fragments was sampled in the same way as in DCSsub (i.e., a combination of lognormal and gamma distributions) and also the distribution into two samples and replicates was achieved as previously described (based on beta and Dirichlet distributions, respectively). The regions together with the information about differential regulation were stored in a bed file, which was then used for the evaluation of the DCS tools. For the complement regions of the input bed file (the regions not listed in the regions bed file), only a simple upper threshold was applied to limit the noise to a user-defined maximum. The input was created by averaging the coverage over all supplied input bam files and then reads were randomly selected. A set of parameter estimation scripts was created to aid the users in defining the parameter settings without creating bam and bed output files but producing informative plots and histograms (https://github.com/Edert/DCSsub).

### Sub-sampling

We used alignment data against chromosome 19 of the mouse reference (mm10) sequence from three independent ChIP-seq experiments: C/EBPa [21] (GSE117780: GSM3308661, GSM3308663 as C/EBPa ChIP-seq data, GSM3308662, GSM3308664 as input control), H3K27ac [24] (GSE158727: GSM4809077, GSM4809078 as H3K27ac ChIP-seq data, GSM4809082, GSM4809081 as input control), and H3K36me3 [25] (GSE110521: GSM2995177, GSM2995178, GSM2995183, GSM2995184 as H3K36me3 ChIP-seq data, GSM2995174, GSM2995171 as input control) for TF, sharp mark and broad mark signals, respectively. Quality control parameters including metrics proposed by the ENCODE [76] and Roadmap Epigenomics [18] consortia and raw sequence read numbers for the genuine ChIP-seq data and the merged files used for DCSsub are shown in Additional file 2: Table S1. QC metrics for the aligned bam files were calculated with ssp (version 1.2.2) [77]. The merged bam files (BEDtools merge) per signal shape in combination with the respective input control bam files and the regions as bed files were used as input for DCSsub. For the region-bed files of C/EBPa and H3K27ac ChIP-seq data, peaks from MACS2 peak calling in mm10 chromosome 19 were filtered for a score bigger than 100 and 130, respectively. For the H3K36me3 regions, peak calling was performed with SICER2 and peaks were filtered for an FDR ≤ 0.01. For each of the DCS scenarios, five sub-sampling runs were performed to obtain five independent datasets. For the C/EBPa ChIP-seq data, we set fragment scaling on and fragment number scaling parameters --frag-count-ex-loc 10 --frag-count-ex-scale 200 were applied. Beta parameters were 0.5|0.5 for the 50:50 and 6.0|0.5 for 100:0 scenarios. The threshold for differential peaks was set to 0.7 with a minimum of 10 reads. The background was set to 0.9. For the H3K27ac ChIP-seq data, we used --frag-count-ex-loc 10 --frag-count-ex-scale 5000 and a background of 0.7. For H3K36me3 data, we applied --frag-count-ex-loc 10 --frag-count-ex-scale 2500 and a background percentage of 0.1.

For the additional genuine ChIP-seq data with lower and higher FRiP, we used data from GSE159627 [43] (GSM4836208, GSM4836209 as STAT6 ChIP-seq data, GSM4836252, GSM4836253 as PU1 ChIP-seq data, GSM4836144, GSM4836145 as H3K4me3 ChIP-seq data and GSM4836077 as input control). H3K9ac ChIP-seq data was used from GSE84314 (Wang Y, Sun Z: Gestational choline supplementation improves cross-generational mood by epigenetic upregulation of Nr3c1, unpublished) (GSM2231391, GSM2231392 as H3K9ac ChIP-seq data and GSM2231389, GSM2231390 as input control), H3K4me3 ChIP-seq data from GSE159627 [43] (GSM4836144, GSM4836145 as H3K4me3 ChIP-seq data and GSM4836077 as input control), H3K27me3 ChIP-seq data from GSE150182 [44] (GSM4542845, GSM4542846 as H3K27me3 ChIP-seq data and GSM4542826, GSM4542828 as input control), and H3K79me2 ChIP-seq data from GSE134083 [45] (GSM3936447, GSM3936448 as H3K79me2 ChIP-seq data and GSM3936504 as input control). For PU1 and STAT6 sub-sampling, we used the same parameters as described above for TF. For STAT6, we set –frag-count-ex-scale to 2500. For H3K4me3 and H3K9ac, we used the same parameters as described for H3K27ac. We sub-sampled H3K27me3 and H3K79me2 as described for H3K36me3.

To sub-sample additional chromosomes, we applied the same parameters as described for C/EBPa, H3K27ac, and H3K36me3 ChIP-seq datasets, and used input control datasets from the same experiments. We adapted the -c parameter according to the chromosome (chr1, chr8, chr11, and chrX), and set the chromosome length accordingly (chr1 195471971, chr8 129401213, chr11 122082543, and chrX 171031299).

### Processing

We used ART [78] (version 2.5.8) on the fasta output files of DCSsim to simulate an Illumina HiSeq 2500 sequencing machine (parameters: art_illumina -ss HS25 -l 50 -c 1 -na). Then the simulated and sub-sampled reads were processed with the same shell-based pipeline.

Quality filtering and trimming, as well as length filtering, was done with PRINSEQ-lite [79] (parameters: -out_bad null -min_len 30 -min_qual_mean 30 -ns_max_n 5 -trim_tail_right 8 -trim_tail_left 8 -trim_qual_right 30 -trim_qual_left 30 -trim_qual_window 5). Alignment against the mouse reference genome sequence (mm10) and alignment of the spike-in data against the *Drosophila melanogaster* genome (dm6_chr2L) was performed with bwa [80] (version 0.7.15-r1140). Results were processed and split into the respective species with samtools [81] (version 1.7). As all reads from the simulation and sub-sampling are meant to stem from the ChIP-seq signal and no additional PCR amplification simulation step was implemented, we kept duplicate single-end reads.

### Signal to noise metrics

We used SSP [77] to calculate the normalized strand coefficient and background uniformity and the plotFingerprint function from deepTools [82] to retrieve the Jensen-Shannon distance and CHANCE divergence for genuine ChIP-seq data, sub-sampled (DCSsub), and simulated (DCSsim) signals.

### Prediction

Peaks were called with JAMM [30] (version 1.0.7rev6), MACS2 [28] (version 2.2.6), and SICER2 [29] (version 1.0) on the simulated and sub-sampled data (Additional file 4: Table S3). JAMM and SICER2 were executed with a sharp and broad setup, resulting in eight peak files per simulated and sub-sampled sample (broad and sharp peak calling for two replicates of samples 1 and 2). For JAMM, the parameters were -r peak and -r window -b 1000 -w 1. SICER2 was applied with window 50, gap 100 and window 100, gap 200, for sharp and broad peak calling respectively. For MACS2, we used default parameters and a -q of 0.05. The --broad option was switched off in one run and on in the second. The --nomodel parameter was set with a --extsize of 200. We used the default -g value that did not influence of the outcome, see Additional file 1: Fig. S1i. This resulted in 4 peak files per comparison and in sum 16 peak files (broad and sharp, model and nomodel, two replicates for samples 1 and 2). Peak-dependent tools were executed with the bam files obtained from simulation and sub-sampling. Each peak calling setup was then included individually. For each DCS tool (all tools and program versions available on September 10, 2020, see Additional file 3: Table S2), we created an individual wrapper shell script, which converts the input bam and bed files into a file format the respective tool requires as input. The time span required by this conversion was saved as preparation time. If required, the virtual environment for the currently tested software was loaded before the tool was applied. After successful prediction, the runtime was stored per parameter setup. The individual parameter setups are shown in Additional file 4: Table S3. Output files were converted into bed format and finally all temporary files were removed. Results were stored in one output bed file with peak positions, their log2 fold-change, and the respective *p*-value, FDR, or score. In addition, a file with runtimes in seconds per parameter setup was created. All tools with the exception of MMDiff2 (extensive memory consumption), ChIPnorm (commercial Matlab software required), GenoGAM, HMCan, and MultiGPS (due to long runtimes, 5 threads were used for these three tools) were run with a single thread on an Intel(R) Xeon(R) CPU E5-2609 v4 @ 1.70 GHz with 64 GB memory. The mentioned exceptions were executed on an Intel(R) Xeon(R) CPU E5-2603 v3 @ 1.60 GHz with 128 GB memory.

### Evaluation

BEDtools [75] was used for preprocessing, and then the R libraries ROCR [83] and flux [84] were applied for curve and AUPRC calculation. In this iterative process, predicted peaks were first sorted according to *p*-value, FDR, or score, depending if lower or higher values represent better results. Then these scores were processed successively, and in each step the true positive, false positive, condition positive, and condition negative bps were calculated and stored. The resulting values were used to create precision-recall curves.

### Random classifier

To compare the AUPRC values from the analysis of DCS tools to values from a random peak prediction, we randomized existing peak data by random shuffling of peak positions or *p*-values and their position and created completely random peak regions with the same mean length (Fig. 3a, Additional file 1: Fig. S3a, Additional file 4: Table S3). To compare the AUPRCs of the benchmarked tools to random region predictions, we created a set of random predictors (Additional file 4: Table S3) that were based on the regions obtained from the simulation and sub-sampling. We applied them on two scenarios and three peak shapes with five replicates each to create dependent random regions, independent random regions, and completely random regions. For the dependent random prediction, we took simulated and sub-sampled regions, assigned randomly shuffled *p*-values, random *p*-values between zero and one, and assigned random *p*-values with the same ratio of changing versus non-changing regions. A random shuffling of the regions inside mm10 chromosome 19 with BEDtools was also performed and data were combined with a 20% increased and 20% decreased region length. For independent random prediction, we created 1000 random regions with the same mean region length of the simulated or sub-sampled template with BEDtools. We then assigned random *p*-values between zero and one and applied the same ratio of changing regions. We did the same for 500 and 2000 simulated regions and for 20% increased and 20% decreased region length. We processed the resulting regions together with the predictions of all DCS tools and used the resulting AUPRCs for comparisons.

### Accuracy profiles

The top 300 predicted differential peaks (i.e., predicted regions sorted by the tool-specific quality metric like *p*-value, FDR, or score) per tool parameter setup were compared to the simulated as well as sub-sampled reference peaks. We chose 300 regions to capture predictions of differential regions only. The minimum number of simulated differential regions was 347. False negatives were calculated by simply summing up all bp in simulated or sub-sampled regions that changed without being predicted. False positives were counted by adding the bp of predicted regions without counterparts in changing regions in the simulated or sub-sampled reference. For the FDR, the number of false positives was divided by the sum of bp of all predicted regions. The FOR was calculated by dividing all false negatives by the sum of not predicted bp, which is the length of chr19 minus the sum of all predicted regions. We subtracted the overlap of individual predictions versus the reference in bp and the number of bp covered by regions not overlapping between these two sets from the total length of all predicted regions to obtain the “too long” measure. Similarly, the “too short” measure was calculated by subtracting the overlap of predictions with true changing regions in bp from the length of all changing reference regions overlapping (≥ 1 bp) with any predicted region in bp. We calculated the percentages for “too long” and “too short” by dividing the values by the length of all simulated or sub-sampled regions in the reference that changed between samples. All region overlaps were determined by BEDtools.

### Stability metrics

As standard deviation, we calculated the standard deviation of the AUPRC between all replicates of a specific tool parameter setup and used the mean of all parameter setups per tool. The number of NA results summarizes all failed runs or runs without output per tool. For the final score calculation, each metric was calculated on parameter setup level.

### DCS score

We combined the AUPRC, the stability metrics (standard deviation and number of NAs), runtime, preparation time and memory consumption for each scenario, tool, and setup combination into one score. We normalized the stability metrics, runtime, preparation time, and memory values via the R library effect size [85] to a range of 0 to 1. For the final score, we summed up the metrics using the following weights: AUPRC 1.6, standard deviation and number of NAs 0.2, runtime 0.4, preparation time 0.2, and memory consumption 0.4.

### Heatmaps, scatter plots, and coverage visualization

We applied deepTools [82] bamCoverage to generate bigWig files and bamCoverage in combination with computeMatrix and plotHeatmap to create the ChIP-seq heatmaps. The combined heatmap of all AUPRCs, accuracy profiles, stability metrics, runtime, and memory consumption was created with the R library ComplexHeatmap [86]. As we inverted the range (1-value) for the normalized stability metrics, runtime, preparation time, and memory consumption, a low value means good performance. For all other bar-, box-, and scatter plots, we used ggplot2 [87]. For the coverage visualization in combination with predicted DCS regions, we applied trackViewer [88].

### Statistical analysis

All statistical analysis was done in R. For the log2 fold-change between simulation and sub-sampling AUPRCs, we first used the Shapiro-Wilk test for normality. As the null hypothesis (normal distribution) was rejected, we applied the two-sided Wilcoxon rank sum test.

## Supplementary Information

**Additional file 1: Figure S1.** Simulation and sub-sampling details. **Figure S2.** AUPRCs of DCS tools based on simulated, sub-sampled, or merged data. **Figure S3.** Assessment of DCS tools per peak shape and regulation scenario based on AUPRCs. **Figure S4.** Coverage and top tool predictions of example DCS regions. **Figure S5.** Signal-to-noise ratio affects AUPRC. **Figure S6.** Effects of chromosome characteristics and ChIP-seq signal distribution on AUPRC. **Figure S7.** Preparation time and runtime plus memory requirements based on individual scenarios. **Figure S8.** Performance measures and ranking of DCS tools per scenario.

**Additional file 2: Table S1.** QC metrics of genuine ChIP-seq data. Table of QC metrics of publicly available ChIP-seq datasets used for sub-sampling with DCSsub. The first six columns represent information on the original sample names, type (if genuine data or merged for subsequent sub-sampling), their peak shape, the replicate, and sample numbers and the regulation scenario in the original experiments respectively. The next columns show the total read number, the nonredundant read number, read length, and estimated fragment length. The QC metrics consist of the normalized strand coefficient (NSC) and the relative strand correlation (RSC) from cross-correlation analysis as signal-to-noise indicator, the background uniformity as a measure for peak reliability (by looking at the distribution of the mapped reads), and the fragment cluster score (FCS) for various strand shifts (read, fragment, 1k, 10k and 100k) to identify peak mode and intensity.

**Additional file 3: Table S2.** Benchmarked DCS tools. Table of DCS tools, their focus, platform / programming language, version used, peak dependence, ease of installation, publication, number of citations (Google Scholar 2019-11-28), and the link to the respective website or download location. Ease of installation: +++ : Execution of a few predefined lines, including all tools that can be installed via Bioconductor and pip. Installation time, seconds-minutes. ++ : Compilation/manual installation of requirements. Installation time, minutes. + : Compilation and manual installation of requirements. Installation time, minutes-hours. - : Manual compilation/installation of source code and requirements or modification in source code required. Installation time, hours-days.

**Additional file 4: Table S3.** Applied parameter setups per DCS tool and peak caller. Table describing DCS tools, peak caller and the random classifier by their name, peak dependence, parameter setup name, if a scaling factor was used, setup description, and setup parameter details for the essential core steps.

**Additional file 5: Table S4.** DCS scores. Table of test scenarios, DCS tools, applied peak caller and parameters, AUPRC values, the scaled runtime and preparation time, scaled memory usage, scaled number of NAs and standard deviation, and the DCS score.

**Additional file 6: Table S5.** DCS scores per peak and regulation scenario. Table of DCS score ranked DCS tools, the applied peak caller and parameter setup, and the DCS score for: all test sets averaged, the 50:50, and 100:0 regulation scenarios, TF, sharp mark, and broad mark peak shapes, and all combinations of regulation scenario, and peak shape: TF 50:50, TF 100:0, sharp mark 50:50, sharp mark 100:0, broad mark 50:50, and broad mark 100:0.

**Additional file 7.** Review history.

## Data Availability

Simulated and sub-sampled data of the six initial test scenarios are available under 10.5281/zenodo.5005654. We used the following publicly available datasets for sub-sampling and the parameter estimation for the simulation: C/EBPa ChIP-seq data from Schmidt, L., Heyes, E., Scheiblecker, L., Eder, T., Volpe, G., Frampton, J., Nerlov, C., Valent, P., Grembecka, J., & Grebien, F.. CEBPA-mutated leukemia is sensitive to genetic and pharmacological targeting of the MLL1 complex. GSE117780. 10.1038/s41375-019-0382-3 (2019).: GSM3308661 C18_shRen_HD3_1 and GSM3308663 C18_shRen_HD3_2 as C/EBPa ChIP-seq data and GSM3308662 INPUT_shRen_HD3_1 and GSM3308664 INPUT_shRen_HD3_2 as input control. H3K27ac ChIP-seq data from Heyes, E., Schmidt, L., Manhart, G., Eder, T., Proietti, L., & Grebien, F.. Identification of gene targets of mutant C/EBPα reveals a critical role for MSI2 in CEBPA-mutated AML. GSE158727. 10.1038/s41375-021-01169-6 (2021).: GSM4809077 H3K27ac_shRen_1 and GSM4809078 H3K27ac_shRen_2 as H3K27ac ChIP-seq data and GSM4809081 H3K27ac_shRen_IN and GSM4809082 H3K27ac_shCebpa_IN as input control. H3K36me3 ChIP-seq data from Skucha, A., Ebner, J., Schmöllerl, J., Roth, M., Eder, T., César-Razquin, A., Stukalov, A., Vittori, S., Muhar, M., Lu, B., Aichinger, M., Jude, J., Müller, A. C., Győrffy, B., Vakoc, C. R., Valent, P., Bennett, K. L., Zuber, J., Superti-Furga, G., & Grebien, F.. MLL-fusion-driven leukemia requires SETD2 to safeguard genomic integrity. GSE110521. 10.1038/s41467-018-04329-y (2018).: GSM2995177 Ren_H3K36me3_1, GSM2995178 Ren_H3K36me3_2, GSM2995183 Setd2_H3K36me3_1 and GSM2995184 Setd2_H3K36me3_2 as H3K36me3 ChIP-seq data and GSM2995174 Setd2_H3K36me3_IN and GSM2995171 Ren_H3K36me3_IN as input control. The following publicly available datasets were used for the comparison of different FRiP and similar shape types with DCSsub: STAT6 and PU1 ChIP-seq data from Hoeksema, M. A., Shen, Z., Holtman, I. R., Zheng, A., Spann, N. J., Cobo, I., Gymrek, M., & Glass, C. K.. Mechanisms underlying divergent responses of genetically distinct macrophages to IL-4. GSE159627. 10.1126/sciadv.abf9808 (2021).: GSM4836208 ChIP_STAT6_C57_BMDM_basal_rep1 and GSM4836209 ChIP_STAT6_C57_BMDM_basal_rep2 as STAT6 ChIP-seq data, GSM4836252 ChIP_PU1_C57_BMDM_basal_rep1 and GSM4836253 ChIP_PU1_C57_BMDM_basal_rep2 as PU1 ChIP-seq data and GSM4836077 ChIP_input_C57_BMDM_basal as input control. H3K9ac ChIP-seq data from Wang Y., Sun Z.. Gestational choline supplementation improves cross-generational mood by epigenetic upregulation of Nr3c1. GSE84314. unpublished (2022): GSM2231391 3030-H3K9_ChIP-seq and GSM2231392 3031-H3K9_ChIP-seq as H3K9ac ChIP-seq data and GSM2231389 3028-INPUT_ChIP-seq and GSM2231390 3029-INPUT_ChIP-seq as input control. H3K4me3 ChIP-seq data from Hoeksema, M. A., Shen, Z., Holtman, I. R., Zheng, A., Spann, N. J., Cobo, I., Gymrek, M., & Glass, C. K.. Mechanisms underlying divergent responses of genetically distinct macrophages to IL-4. GSE159627. 10.1126/sciadv.abf9808 (2021).: GSM4836144 ChIP_H3K4me3_C57_BMDM_basal_rep1 and GSM4836145 ChIP_H3K4me3_C57_BMDM_basal_rep2 as H3K4me3 ChIP-seq data and GSM4836077 ChIP_input_C57_BMDM_basal as input control. H3K27me3 ChIP-seq data from Hota, S. K., Rao, K. S., Blair, A. P., Khalilimeybodi, A., Hu, K. M., Thomas, R., So, K., Kameswaran, V., Xu, J., Polacco, B. J., Desai, R. V., Chatterjee, N., Hsu, A., Muncie, J. M., Blotnick, A. M., Winchester, S., Weinberger, L. S., Hüttenhain, R., Kathiriya, I. S., Krogan, N. J., … Bruneau, B. G.. Brahma safeguards canalization of cardiac mesoderm differentiation. GSE150182. 10.1038/s41586-021-04336-y (2022).: GSM4542845 D6_WT_H3K27me3_ChIP_rep1 and GSM4542846 D6_WT_H3K27me3_ChIP_rep2 as H3K27me3 ChIP-seq data and GSM4542826 D6_WT_Input_rep1 and GSM4542828 D6_WT_Input_rep2 as input control. H3K79me2 ChIP-seq data from Cao, K., Ugarenko, M., Ozark, P. A., Wang, J., Marshall, S. A., Rendleman, E. J., Liang, K., Wang, L., Zou, L., Smith, E. R., Yue, F., & Shilatifard, A.. DOT1L-controlled cell-fate determination and transcription elongation are independent of H3K79 methylation. GSE134083. 10.1073/pnas.2001075117 (2020).: GSM3936447 WT_H3K79me2_1 and GSM3936448 WT_H3K79me2_2 as H3K79me2 ChIP-seq data and GSM3936504 WT input as input control. Simulated and sub-sampled FRiP data are available under: 10.5281/zenodo.6042902 and the data with additional chromosomes under: 10.5281/zenodo.6043477. The source code for DCSsim is available under https://github.com/Edert/DCSsim, DOI: 10.5281/zenodo.5118511 and for DCSsub under https://github.com/Edert/DCSsub, DOI: 10.5281/zenodo.5118515. All DCS tools wrapper scripts can be accessed at https://github.com/Edert/DCS_predictions, DOI: 10.5281/zenodo.5118521 and https://github.com/Edert/DCS_predictions_chr, DOI: 10.5281/zenodo.6043882 for the wrapper scripts dealing with other chromosomes than chr19.

## References

[1] Johnson DS, Mortazavi A, Myers RM, Wold B (2007). Genome-wide mapping of in vivo protein-DNA interactions. Science.

[2] Robertson G, Hirst M, Bainbridge M, Bilenky M, Zhao Y, Zeng T (2007). Genome-wide profiles of STAT1 DNA association using chromatin immunoprecipitation and massively parallel sequencing. Nat Methods.

[3] Xie W, Schultz MD, Lister R, Hou Z, Rajagopal N, Ray P (2013). Epigenomic analysis of multilineage differentiation of human embryonic stem cells. Cell.

[4] Zhu J, Adli M, Zou JY, Verstappen G, Coyne M, Zhang X (2013). Genome-wide chromatin state transitions associated with developmental and environmental cues. Cell.

[5] Ernst J, Kheradpour P, Mikkelsen TS, Shoresh N, Ward LD, Epstein CB (2011). Mapping and analysis of chromatin state dynamics in nine human cell types. Nature.

[6] Mikkelsen TS, Ku M, Jaffe DB, Issac B, Lieberman E, Giannoukos G (2007). Genome-wide maps of chromatin state in pluripotent and lineage-committed cells. Nature.

[7] Lara-Astiaso D, Weiner A, Lorenzo-Vivas E, Zaretsky I, Jaitin DA, David E (2014). Chromatin state dynamics during blood formation. Science.

[8] Wang W, Hu CK, Zeng A, Alegre D, Hu D, Gotting K (2020). Changes in regeneration-responsive enhancers shape regenerative capacities in vertebrates. Science.

[9] Jorstad NL, Wilken MS, Grimes WN, Wohl SG, VandenBosch LS, Yoshimatsu T (2017). Stimulation of functional neuronal regeneration from Müller glia in adult mice. Nature.

[10] Zhao Z, Shilatifard A (2019). Epigenetic modifications of histones in cancer. Genome Biol.

[11] Grosselin K, Durand A, Marsolier J, Poitou A, Marangoni E, Nemati F (2019). High-throughput single-cell ChIP-seq identifies heterogeneity of chromatin states in breast cancer. Nat Genet.

[12] Stelloo S, Nevedomskaya E, Kim Y, Schuurman K, Valle-Encinas E, Lobo J (2018). Integrative epigenetic taxonomy of primary prostate cancer. Nat Commun.

[13] Farh KKH, Marson A, Zhu J, Kleinewietfeld M, Housley WJ, Beik S (2015). Genetic and epigenetic fine mapping of causal autoimmune disease variants. Nature.

[14] Soskic B, Cano-Gamez E, Smyth DJ, Rowan WC, Nakic N, Esparza-Gordillo J (2019). Chromatin activity at GWAS loci identifies T cell states driving complex immune diseases. Nat Genet.

[15] Pilon AM, Ajay SS, Kumar SA, Steiner LA, Cherukuri PF, Wincovitch S (2011). Genome-wide ChIP-Seq reveals a dramatic shift in the binding of the transcription factor erythroid Kruppel-like factor during erythrocyte differentiation. Blood.

[16] Orlando DA, Chen MW, Brown VE, Solanki S, Choi YJ, Olson ER (2014). Quantitative ChIP-Seq normalization reveals global modulation of the epigenome. Cell Rep.

[17] Wu DY, Bittencourt D, Stallcup MR, Siegmund KD (2015). Identifying differential transcription factor binding in ChIP-seq. Front Genet.

[18] Kundaje A, Meuleman W, Ernst J, Bilenky M, Yen A, Roadmap Epigenomics Consortium (2015). Integrative analysis of 111 reference human epigenomes. Nature.

[19] Pepke S, Wold B, Mortazavi A (2009). Computation for ChIP-seq and RNA-seq studies. Nat Methods.

[20] Nakato R, Sakata T (2021). Methods for ChIP-seq analysis: a practical workflow and advanced applications. Methods.

[21] Schmidt L, Heyes E, Scheiblecker L, Eder T, Volpe G, Frampton J (2019). CEBPA-mutated leukemia is sensitive to genetic and pharmacological targeting of the MLL1 complex. Leukemia.

[22] Fasan A, Haferlach C, Alpermann T, Jeromin S, Grossmann V, Eder C (2014). The role of different genetic subtypes of CEBPA mutated AML. Leukemia.

[23] Zhang Y, Wang F, Chen X, Liu W, Fang J, Wang M (2019). Mutation profiling of 16 candidate genes in de novo acute myeloid leukemia patients. Front Med.

[24] Heyes E, Schmidt L, Manhart G, Eder T, Proietti L, Grebien F (2021). Identification of gene targets of mutant C/EBPα reveals a critical role for MSI2 in CEBPA-mutated AML. Leukemia.

[25] Skucha A, Ebner J, Schmöllerl J, Roth M, Eder T, César-Razquin A, et al. MLL-fusion-driven leukemia requires SETD2 to safeguard genomic integrity. Nat Commun. 2018;9(1). 10.1038/s41467-018-04329-y PMC5959866 29777171

[26] Heintzman ND, Hon GC, Hawkins RD, Kheradpour P, Stark A, Harp LF (2009). Histone modifications at human enhancers reflect global cell-type-specific gene expression. Nature.

[27] Gates LA, Foulds CE, O’Malley BW (2017). Histone marks in the ‘driver’s seat’: functional roles in steering the transcription cycle. Trends Biochem Sci.

[28] Zhang Y, Liu T, Meyer CA, Eeckhoute J, Johnson DS, Bernstein BE (2008). Model-based analysis of ChIP-Seq (MACS). Genome Biol.

[29] Zang C, Schones DE, Zeng C, Cui K, Zhao K, Peng W (2009). A clustering approach for identification of enriched domains from histone modification ChIP-Seq data. Bioinformatics.

[30] Ibrahim MM, Lacadie SA, Ohler U (2015). JAMM: a peak finder for joint analysis of NGS replicates. Bioinformatics.

[31] Stricker G, Galinier M, Gagneur J. GenoGAM 2.0: scalable and efficient implementation of genome-wide generalized additive models for gigabase-scale genomes. BMC Bioinformatics. 2018;19(1). 10.1186/s12859-018-2238-7 PMC6020310 29945559

[32] Lun ATL, Smyth GK (2016). csaw: a Bioconductor package for differential binding analysis of ChIP-seq data using sliding windows. Nucleic Acids Res.

[33] Mateos JL, Madrigal P, Tsuda K, Rawat V, Richter R, Romera-Branchat M, et al. Combinatorial activities of SHORT VEGETATIVE PHASE and FLOWERING LOCUS C define distinct modes of flowering regulation in Arabidopsis. Genome Biol. 2015;16(1). 10.1186/s13059-015-0597-1 PMC4378019 25853185

[34] Lienhard M, Grimm C, Morkel M, Herwig R, Chavez L (2014). MEDIPS: genome-wide differential coverage analysis of sequencing data derived from DNA enrichment experiments. Bioinformatics.

[35] Zhang Y, Lin YH, Johnson TD, Rozek LS, Sartor MA (2014). PePr: a peak-calling prioritization pipeline to identify consistent or differential peaks from replicated ChIP-Seq data. Bioinformatics.

[36] Huang W, Umbach DM, Vincent Jordan N, Abell AN, Johnson GL, Li L (2011). Efficiently identifying genome-wide changes with next-generation sequencing data. Nucleic Acids Res.

[37] Robinson MD, McCarthy DJ, Smyth GK (2010). edgeR: a Bioconductor package for differential expression analysis of digital gene expression data. Bioinformatics.

[38] Stark R, Brown G (2011). DiffBind: differential binding analysis of ChIP-Seq peak data.

[39] Love MI, Huber W, Anders S. Moderated estimation of fold change and dispersion for RNA-seq data with DESeq2. Genome Biol. 2014;15(12). 10.1186/s13059-014-0550-8 PMC4302049 25516281

[40] Heinz S, Benner C, Spann N, Bertolino E, Lin YC, Laslo P (2010). Simple combinations of lineage-determining transcription factors prime cis-regulatory elements required for macrophage and B cell identities. Mol Cell.

[41] Tu S, Li M, Tan F, Chen H, Xu J, Waxman DJ, et al. MAnorm2 for quantitatively comparing groups of ChIP-seq samples. Bioinformatics. 2020. 10.1101/gr.262675.120 PMC7849384 33208455

[42] Landt SG, Marinov GK, Kundaje A, Kheradpour P, Pauli F, Batzoglou S (2012). ChIP-seq guidelines and practices of the ENCODE and modENCODE consortia. Genome Res.

[43] Hoeksema MA, Shen Z, Holtman IR, Zheng A, Spann NJ, Cobo I (2021). Mechanisms underlying divergent responses of genetically distinct macrophages to IL-4. Sci Adv.

[44] Hota SK, Rao KS, Blair AP, Khalilimeybodi A, Hu KM, Thomas R (2022). Brahma safeguards canalization of cardiac mesoderm differentiation. Nature.

[45] Cao K, Ugarenko M, Ozark PA, Wang J, Marshall SA, Rendleman EJ (2020). DOT1L-controlled cell-fate determination and transcription elongation are independent of H3K79 methylation. Proc Natl Acad Sci U S A.

[46] Mahony S, Edwards MD, Mazzoni EO, Sherwood RI, Kakumanu A, Morrison CA (2014). An integrated model of multiple-condition ChIP-Seq data reveals predeterminants of Cdx2 binding. Ioshikhes I, editor. PLoS Comput Biol.

[47] Schweikert G, Kuo D (2019). MMDiff2: statistical testing for ChIP-Seq data sets.

[48] Chen L, Wang C, Qin ZS, Wu H (2015). A novel statistical method for quantitative comparison of multiple ChIP-seq datasets. Bioinformatics.

[49] Taslim C, Huang T, Lin S (2011). DIME: R-package for identifying differential ChIP-seq based on an ensemble of mixture models. Bioinformatics.

[50] Ashoor H, Louis-Brennetot C, Janoueix-Lerosey I, Bajic VB, Boeva V. HMCan-diff: a method to detect changes in histone modifications in cells with different genetic characteristics. Nucleic Acids Res. 2017. 10.1093/nar/gkw1319 PMC5416852 28053124

[51] Song Q, Smith AD (2011). Identifying dispersed epigenomic domains from ChIP-Seq data. Bioinformatics.

[52] Helmuth J, Li N, Arrigoni L, Gianmoena K, Cadenas C, Gasparoni G, et al. normR: regime enrichment calling for ChIP-seq data. bioRxiv. 2016. 10.1101/082263.

[53] Shao Z, Zhang Y, Yuan GC, Orkin SH, Waxman DJ (2012). MAnorm: a robust model for quantitative comparison of ChIP-Seq data sets. Genome Biol.

[54] Xu H, Wei CL, Lin F, Sung WK (2008). An HMM approach to genome-wide identification of differential histone modification sites from ChIP-seq data. Bioinformatics.

[55] Nair NU, Sahu AD, Bucher P, Moret BME (2012). ChIPnorm: a statistical method for normalizing and identifying differential regions in histone modification ChIP-seq libraries. Mariño-Ramírez L, editor. PLoS One.

[56] Taudt A, Nguyen MA, Heinig M, Johannes F, Colome-Tatche M. chromstaR: tracking combinatorial chromatin state dynamics in space and time. bioRxiv. 2016. 10.1101/038612.

[57] Shen L, Shao NY, Liu X, Maze I, Feng J, Nestler EJ (2013). diffReps: detecting differential chromatin modification sites from ChIP-seq data with biological replicates. Mantovani R, editor. PLoS One.

[58] Heinig M, Colomé-Tatché M, Taudt A, Rintisch C, Schafer S, Pravenec M, et al. histoneHMM: differential analysis of histone modifications with broad genomic footprints. BMC Bioinformatics. 2015;16(1). 10.1186/s12859-015-0491-6 PMC4347972 25884684

[59] Allhoff M, Seré K, Chauvistré H, Lin Q, Zenke M, Costa IG (2014). Detecting differential peaks in ChIP-seq signals with ODIN. Bioinformatics.

[60] Liu B, Yi J, Sv A, Lan X, Ma Y, Huang TH (2013). QChIPat: a quantitative method to identify distinct binding patterns for two biological ChIP-seq samples in different experimental conditions. BMC Genomics.

[61] Allhoff M, Seré K, Pires JF, Zenke M, Costa IG. Differential peak calling of ChIP-seq signals with replicates with THOR. Nucleic Acids Res. 2016. 10.1093/nar/gkw680 PMC5175345 27484474

[62] Steinhauser S, Kurzawa N, Eils R, Herrmann C. A comprehensive comparison of tools for differential ChIP-seq analysis. Brief Bioinform. 2016. 10.1093/bib/bbv110 PMC5142015 26764273

[63] Boyle AP, Davis S, Shulha HP, Meltzer P, Margulies EH, Weng Z (2008). High-resolution mapping and characterization of open chromatin across the genome. Cell.

[64] Schones DE, Cui K, Cuddapah S, Roh TY, Barski A, Wang Z (2008). Dynamic regulation of nucleosome positioning in the human genome. Cell.

[65] Gaulton KJ, Nammo T, Pasquali L, Simon JM, Giresi PG, Fogarty MP (2010). A map of open chromatin in human pancreatic islets. Nat Genet.

[66] Steensel BV, Henikoff S (2000). Identification of in vivo DNA targets of chromatin proteins using tethered Dam methyltransferase. Nat Biotechnol.

[67] Greil F, Moorman C, van Steensel B. [16] DamID: mapping of in vivo protein–genome interactions using tethered DNA adenine methyltransferase. In: Methods in enzymology: Elsevier; 2006. p. 342–59. 10.1016/S0076-6879(06)10016-6 16938559

[68] Vogel MJ, Peric-Hupkes D, van Steensel B (2007). Detection of in vivo protein–DNA interactions using DamID in mammalian cells. Nat Protoc.

[69] Skene PJ, Henikoff S (2017). An efficient targeted nuclease strategy for high-resolution mapping of DNA binding sites. eLife.

[70] Buenrostro JD, Giresi PG, Zaba LC, Chang HY, Greenleaf WJ (2013). Transposition of native chromatin for fast and sensitive epigenomic profiling of open chromatin, DNA-binding proteins and nucleosome position. Nat Methods.

[71] Reske JJ, Wilson MR, Chandler RL. ATAC-seq normalization method can significantly affect differential accessibility analysis and interpretation. Epigenetics Chromatin. 2020;13(1). 10.1186/s13072-020-00342-y PMC7178746 32321567

[72] Buenrostro JD, Wu B, Litzenburger UM, Ruff D, Gonzales ML, Snyder MP (2015). Single-cell chromatin accessibility reveals principles of regulatory variation. Nature.

[73] Cusanovich DA, Daza R, Adey A, Pliner HA, Christiansen L, Gunderson KL (2015). Multiplex single-cell profiling of chromatin accessibility by combinatorial cellular indexing. Science.

[74] Rotem A, Ram O, Shoresh N, Sperling RA, Goren A, Weitz DA (2015). Single-cell ChIP-seq reveals cell subpopulations defined by chromatin state. Nat Biotechnol.

[75] Quinlan AR, Hall IM (2010). BEDTools: a flexible suite of utilities for comparing genomic features. Bioinformatics.

[76] The ENCODE Project Consortium (2012). An integrated encyclopedia of DNA elements in the human genome. Nature.

[77] Nakato R, Shirahige K (2018). Sensitive and robust assessment of ChIP-seq read distribution using a strand-shift profile. Birol I, editor. Bioinformatics.

[78] Huang W, Li L, Myers JR, Marth GT (2012). ART: a next-generation sequencing read simulator. Bioinformatics.

[79] Schmieder R, Edwards R (2011). Quality control and preprocessing of metagenomic datasets. Bioinformatics.

[80] Li H, Durbin R (2009). Fast and accurate short read alignment with Burrows-Wheeler transform. Bioinformatics.

[81] Li H, Handsaker B, Wysoker A, Fennell T, Ruan J, Homer N (2009). The Sequence Alignment/Map format and SAMtools. Bioinformatics.

[82] Ramirez F, Dundar F, Diehl S, Gruning BA, Manke T (2014). deepTools: a flexible platform for exploring deep-sequencing data. Nucleic Acids Res.

[83] Sing T, Sander O, Beerenwinkel N, Lengauer T (2005). ROCR: visualizing classifier performance in R. Bioinformatics.

[84] Jurasinski G, Koebsch F, Guenther A, Beetz S (2014). flux: flux rate calculation from dynamic closed chamber measurements.

[85] Ben-Shachar M, Lüdecke D, Makowski D (2020). effectsize: estimation of effect size indices and standardized parameters. JOSS.

[86] Gu Z, Eils R, Schlesner M (2016). Complex heatmaps reveal patterns and correlations in multidimensional genomic data. Bioinformatics.

[87] Wickham H (2016). ggplot2: elegant graphics for data analysis.

[88] Ou J, Zhu LJ (2019). trackViewer: a Bioconductor package for interactive and integrative visualization of multi-omics data. Nat Methods.

