# Supplementary material for: Comprehensive assessment of differential ChIP-seq tools guides optimal algorithm selection
Source: Genome Biol. 2022 May 24;23:119. doi: 10.1186/s13059-022-02686-y (PMC9128273; doi:10.1186/s13059-022-02686-y)
Supplement: Supplementary file 1 — Additional file 1: Figure S1. Simulation and sub-sampling details. Figure S2. AUPRCs of DCS tools based on simulated, sub-sampled, or merged data. Figure S3. Assessment of DCS tools per peak shape and regulation scenario based on AUPRCs. Figure S4. Coverage and top tool predictions of example DCS regions. Figure S5. Signal-to-noise ratio affects AUPRC. Figure S6. Effects of chromosome characteristics and ChIP-seq signal distribution on AUPRC. Figure S7. Preparation time and runtime plus memory requirements based on individual scenarios. Figure S8. Performance measures and ranking of DCS tools per scenario. [file 13059_2022_2686_MOESM1_ESM.pdf]

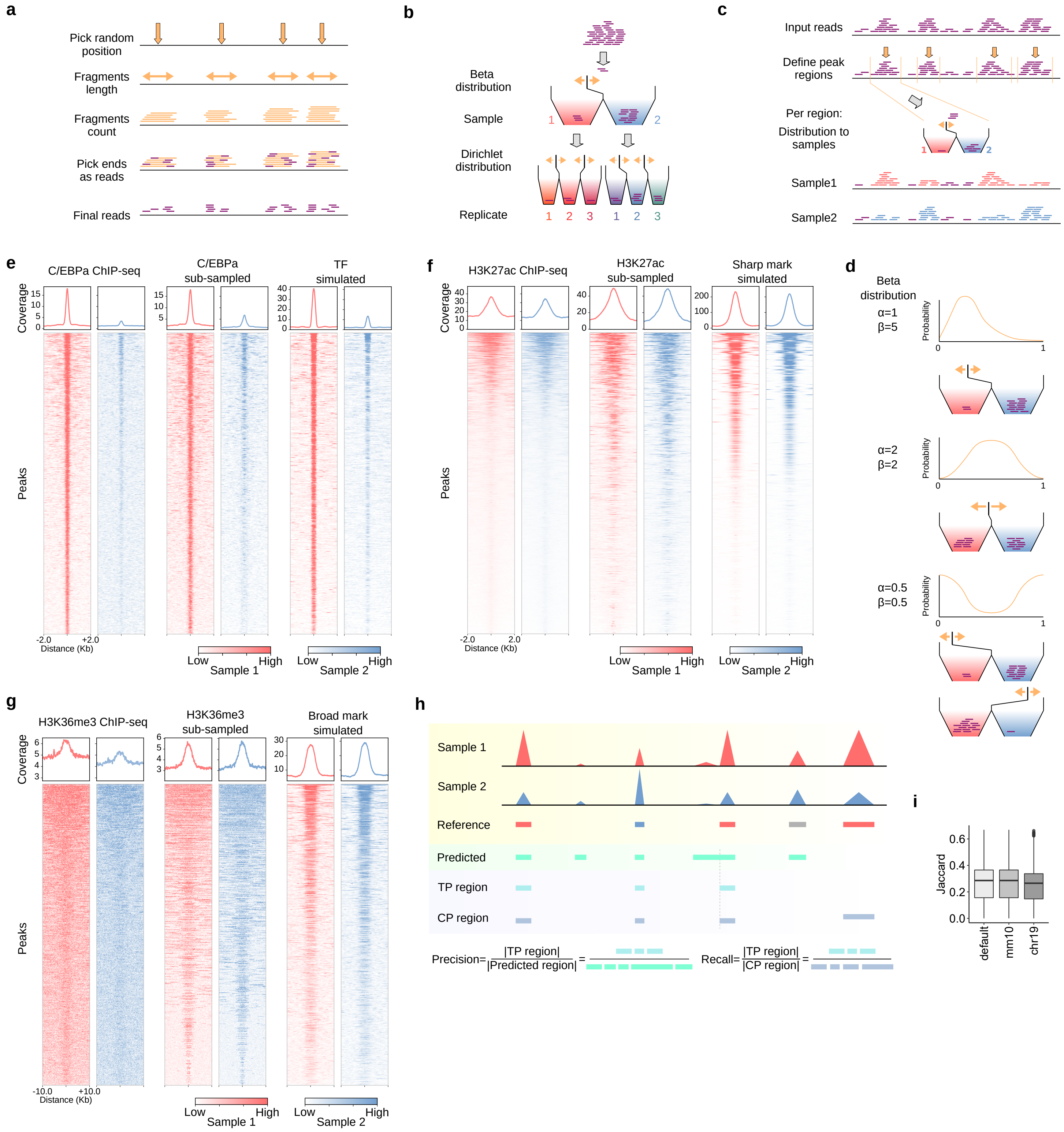
a
Pick random position
Fragments length
Fragments count
Pick ends as reads
Final reads
b
Beta distribution
Sample
Dirichlet distribution
Replicate
c
Input reads
Define peak regions
Per region:
Distribution to samples
Sample1
Sample2
d
Beta distribution
α=1
β=5
α=2
β=2
α=0.5
β=0.5
Probability
e
C/EBPa ChIP-seq
C/EBPa sub-sampled
TF simulated
Coverage
Peaks
Distance (Kb)
Low
High
Sample 1
Sample 2
f
H3K27ac ChIP-seq
H3K27ac sub-sampled
Sharp mark simulated
g
H3K36me3 ChIP-seq
H3K36me3 sub-sampled
Broad mark simulated
h
Sample 1
Sample 2
Reference
Predicted
TP region
CP region
Precision= |TP region| / |Predicted region|
Recall= |TP region| / |CP region|
i
Jaccard
default
mm10
chr19

Figure S1: **Simulation and sub-sampling details**

a, Overview of DCSsim *in-silico* simulation. A defined number of random positions was picked within the provided genome sequence. The individual fragment length and the number of fragments per position were drawn from respective distributions (see methods). For the final reads, 3’ or 5’ ends of the fragments were randomly selected. **b**, Reads were distributed to samples and replicates via beta- and Dirichlet distributions, respectively. **c**, Scheme of DCSsub sub-sampling. Starting from aligned sequence reads, reads were distributed to samples and replicates as shown in b. This was done for all predefined peak regions indicated by orange arrows. The reads outside these regions, reduced by a user defined percentage, were randomly distributed to the two samples. **d**, Details of the gating strategy. Per region, the position of the gate was drawn from a beta distribution defined by the alpha and beta values. Alpha 1 and beta 5: high probability of sorting most reads into sample 2 (top). Alpha 2 and beta 2: most cases result in a nearly equal number of reads in both samples (middle). Alpha and beta of 0.5: the majority of reads sorted to either sample 1 or sample 2 (bottom). **e**, Heatmaps and profile plots for all peak regions of ChIP-seq experiments for the TF C/EBPa and after its knockdown (left), DCSsub sub-sampling of a 100:0 scenario from this data (middle) and the simulation with DCSsim for TF peak shape and 100:0 regulation (right). **f**, Heatmaps and profile plots from genuine ChIP-seq data (left), DCSsub sub-sampled (middle) and DCSsim simulated data (right) from an H3K27ac histone mark ChIP-seq experiment before and after perturbation of C/EBPa, with a 50:50 regulation scenario. **g**, Heatmaps and profile plots from genuine ChIP-seq data (left), DCSsub sub-sampled (middle) and DCSsim simulated data (right) from an H3K36me3 histone mark ChIP-seq experiment before and after perturbation of SETD2. **h**, Schematic representation of the evaluation procedure. Reference regions were compared to the predictions from the DCS tools. Colors of the reference regions indicate the origin of the higher signal (red sample 1 and blue sample 2). True positive (TP) and condition positive (CP) regions were used to calculate precision and recall. **j**, Influence of the MACS2 -g parameter. Boxplots resemble the Jaccard similarity between the resulting bed files and the simulated reference (default: 2.7e9, mm10: 1.87e9 and mm10 chr19: 5,8205,856).

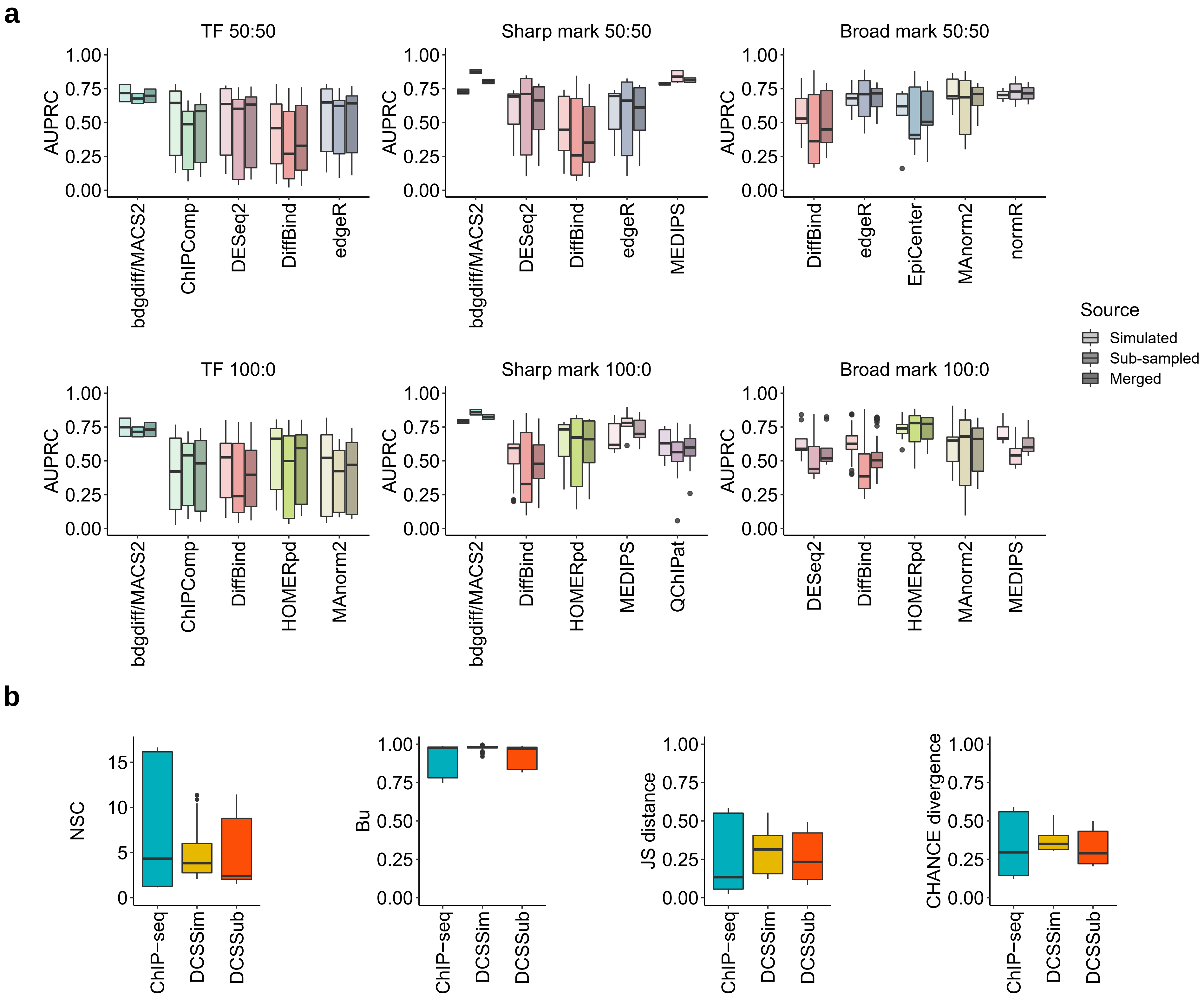

a
TF 50:50
Sharp mark 50:50
Broad mark 50:50
AUPRC
bdgdiff/MACS2
ChIPComp
DESeq2
DiffBind
edgeR
MEDIPS
EpiCenter
MAnorm2
normR
TF 100:0
Sharp mark 100:0
Broad mark 100:0
HOMERpd
QChIPat
Source
Simulated
Sub-sampled
Merged
b
NSC
Bu
JS distance
CHANCE divergence
ChIP−seq
DCSSim
DCSSub

Figure S2: **AUPRCs of DCS tools based on simulated, sub-sampled or merged data**

a, AUPRCs of the best performing tools per test scenario for simulated or sub-sampled data only and the merged AUPRCs. Rows showing TFs (left), sharp (middle) and broad marks (right) and the columns 50:50 (top) and 100:0 (bottom) regulation. Box plot limits, 25% and 75% quantiles; center line, median; whiskers, 1.5x interquartile range. **b**, Assessment of signal-to-noise metrics is shown by normalized strand coefficient (NSC), background uniformity (Bu), Jensen-Shannon (JS) distance and CHANCE divergence for real, DCSsim and DCSsub data.

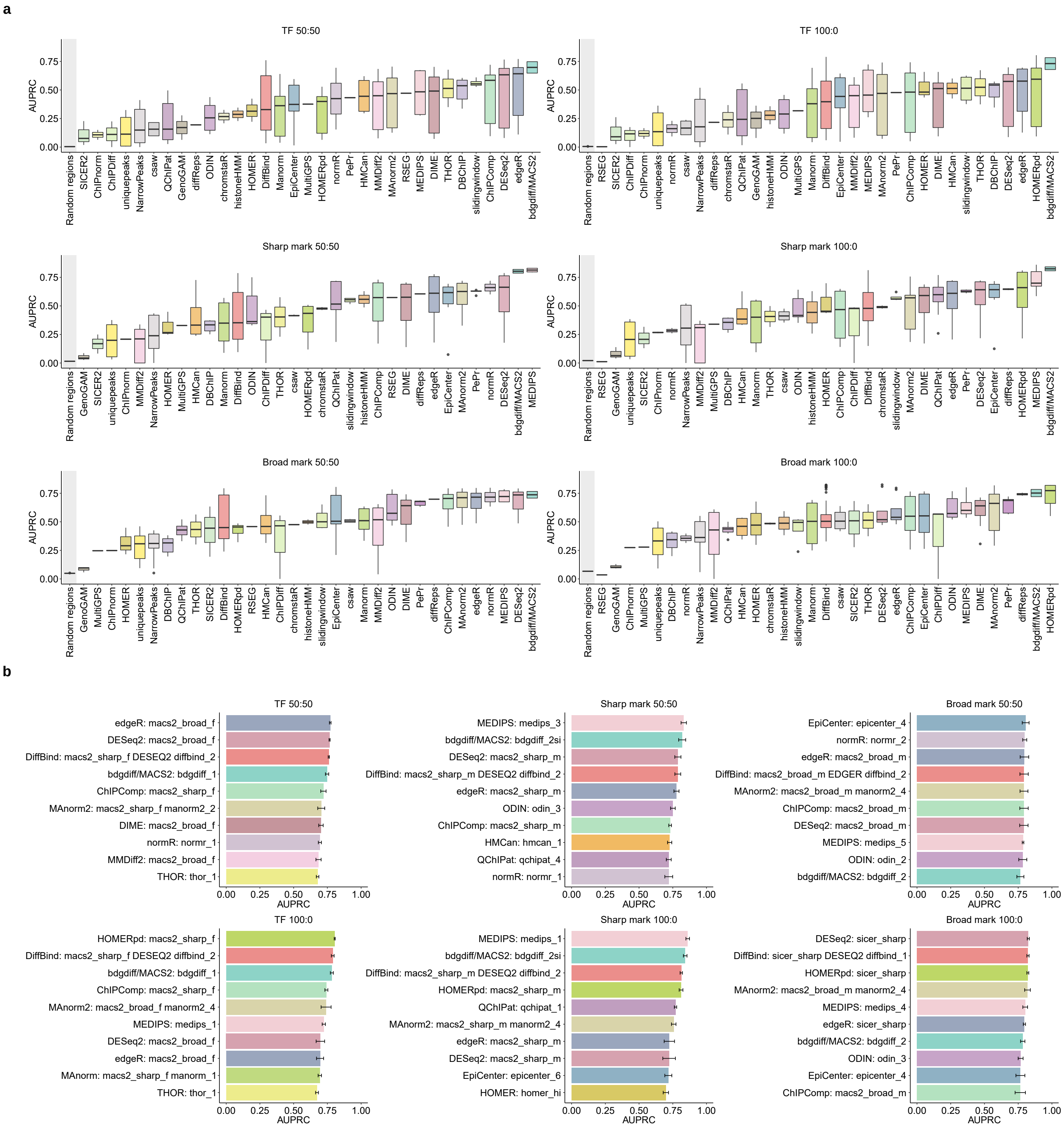
a
TF 50:50
AUPRC
0.00
0.25
0.50
0.75
Random regions
SICER2
ChIPnorm
ChIPDiff
uniquepeaks
NarrowPeaks
csaw
QChIPat
GenoGAM
diffReps
ODIN
chromstaR
histoneHMM
HOMER
DiffBind
Manorm
EpiCenter
MultiGPS
HOMERpd
normR
PePr
HMCan
MMDiff2
MAnorm2
RSEG
MEDIPS
DIME
THOR
DBChIP
slidingwindow
ChIPComp
DESeq2
edgeR
bdgdiff/MACS2
TF 100:0
Random regions
RSEG
SICER2
ChIPDiff
ChIPnorm
uniquepeaks
normR
csaw
NarrowPeaks
diffReps
chromstaR
QChIPat
GenoGAM
histoneHMM
ODIN
MultiGPS
Manorm
DiffBind
EpiCenter
MMDiff2
MEDIPS
MAnorm2
PePr
ChIPComp
HOMER
DIME
HMCan
slidingwindow
THOR
DBChIP
DESeq2
edgeR
HOMERpd
bdgdiff/MACS2
Sharp mark 50:50
Random regions
GenoGAM
SICER2
uniquepeaks
ChIPnorm
MMDiff2
NarrowPeaks
HOMER
MultiGPS
HMCan
DBChIP
Manorm
DiffBind
ODIN
ChIPDiff
THOR
csaw
HOMERpd
chromstaR
QChIPat
slidingwindow
histoneHMM
ChIPComp
RSEG
DIME
diffReps
edgeR
EpiCenter
MAnorm2
PePr
normR
DESeq2
bdgdiff/MACS2
MEDIPS
Sharp mark 100:0
Random regions
RSEG
GenoGAM
uniquepeaks
SICER2
ChIPnorm
normR
csaw
NarrowPeaks
MMDiff2
MultiGPS
DBChIP
HMCan
Manorm
THOR
csaw
ODIN
histoneHMM
HOMER
ChIPComp
ChIPDiff
DiffBind
chromstaR
slidingwindow
MAnorm2
DIME
QChIPat
edgeR
PePr
DESeq2
EpiCenter
diffReps
HOMERpd
MEDIPS
bdgdiff/MACS2
Broad mark 50:50
Random regions
GenoGAM
MultiGPS
ChIPnorm
HOMER
uniquepeaks
NarrowPeaks
DBChIP
QChIPat
THOR
SICER2
DiffBind
HOMERpd
RSEG
HMCan
ChIPDiff
chromstaR
histoneHMM
slidingwindow
EpiCenter
csaw
Manorm
MMDiff2
ODIN
DIME
PePr
diffReps
ChIPComp
MAnorm2
edgeR
normR
MEDIPS
DESeq2
bdgdiff/MACS2
Broad mark 100:0
Random regions
RSEG
GenoGAM
ChIPnorm
MultiGPS
uniquepeaks
DBChIP
normR
NarrowPeaks
MMDiff2
QChIPat
HMCan
HOMER
chromstaR
histoneHMM
slidingwindow
Manorm
DiffBind
csaw
SICER2
THOR
DESeq2
edgeR
ChIPComp
EpiCenter
ChIPDiff
ODIN
MEDIPS
DIME
MAnorm2
PePr
diffReps
bdgdiff/MACS2
HOMERpd
b
TF 50:50
edgeR: macs2_broad_f
DESeq2: macs2_broad_f
DiffBind: macs2_sharp_f DESEQ2 diffbind_2
bdgdiff/MACS2: bdgdiff_1
ChIPComp: macs2_sharp_f
MAnorm2: macs2_sharp_f manorm2_2
DIME: macs2_broad_f
normR: normr_1
MMDiff2: macs2_broad_f
THOR: thor_1
0.00 0.25 0.50 0.75 1.00
AUPRC
Sharp mark 50:50
MEDIPS: medips_3
bdgdiff/MACS2: bdgdiff_2si
DESeq2: macs2_sharp_m
DiffBind: macs2_sharp_m DESEQ2 diffbind_2
edgeR: macs2_sharp_m
ODIN: odin_3
ChIPComp: macs2_sharp_m
HMCan: hmcan_1
QChIPat: qchipat_4
normR: normr_1
Broad mark 50:50
EpiCenter: epicenter_4
normR: normr_2
edgeR: macs2_broad_m
DiffBind: macs2_broad_m EDGER diffbind_2
MAnorm2: macs2_broad_m manorm2_4
ChIPComp: macs2_broad_m
DESeq2: macs2_broad_m
MEDIPS: medips_5
ODIN: odin_2
bdgdiff/MACS2: bdgdiff_2
TF 100:0
HOMERpd: macs2_sharp_f
DiffBind: macs2_sharp_f DESEQ2 diffbind_2
bdgdiff/MACS2: bdgdiff_1
ChIPComp: macs2_sharp_f
MAnorm2: macs2_broad_f manorm2_4
MEDIPS: medips_1
DESeq2: macs2_broad_f
edgeR: macs2_broad_f
MAnorm: macs2_sharp_f manorm_1
THOR: thor_1
Sharp mark 100:0
MEDIPS: medips_1
bdgdiff/MACS2: bdgdiff_2si
DiffBind: macs2_sharp_m DESEQ2 diffbind_2
HOMERpd: macs2_sharp_m
QChIPat: qchipat_1
MAnorm2: macs2_sharp_m manorm2_4
edgeR: macs2_sharp_m
DESeq2: macs2_sharp_m
EpiCenter: epicenter_6
HOMER: homer_hi
Broad mark 100:0
DESeq2: sicer_sharp
DiffBind: sicer_sharp DESEQ2 diffbind_1
HOMERpd: sicer_sharp
MAnorm2: macs2_broad_m manorm2_4
MEDIPS: medips_4
edgeR: sicer_sharp
bdgdiff/MACS2: bdgdiff_2
ODIN: odin_3
EpiCenter: epicenter_4
ChIPComp: macs2_broad_m

Figure S3: **Assessment of DCS tools per peak shape and regulation scenario based on AUPRCs**

a, AUPRC values of DCS tools for each of the six shape and regulation scenarios ranked by median AUPRC plus the respective random predictions on the left (gray background). Box plot limits, 25% and 75% quantiles; center line, median; whiskers, 1.5x interquartile range. **b**, AUPRCs of the respective best parameter setups for the top ten DCS tools per scenario. Labels indicate DCS tool and peak caller and/or parameter setup identifier (for details see Table S3) if applicable; whiskers, standard error of the mean.

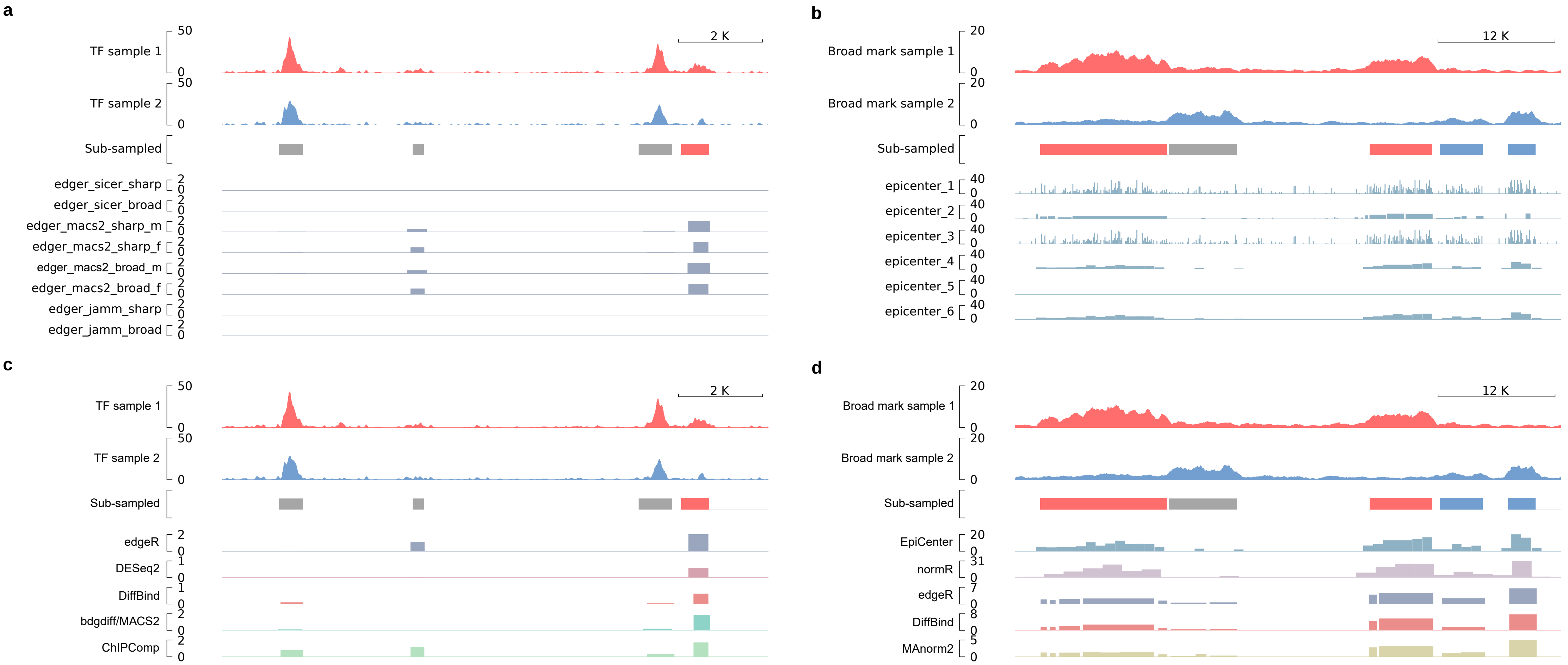

a
TF sample 1
TF sample 2
Sub-sampled
edger_sicer_sharp
edger_sicer_broad
edger_macs2_sharp_m
edger_macs2_sharp_f
edger_macs2_broad_m
edger_macs2_broad_f
edger_jamm_sharp
edger_jamm_broad
2 K
b
Broad mark sample 1
Broad mark sample 2
Sub-sampled
epicenter_1
epicenter_2
epicenter_3
epicenter_4
epicenter_5
epicenter_6
12 K
c
TF sample 1
TF sample 2
Sub-sampled
edgeR
DESeq2
DiffBind
bdgdiff/MACS2
ChIPComp
2 K
d
Broad mark sample 1
Broad mark sample 2
Sub-sampled
EpiCenter
normR
edgeR
DiffBind
MAnorm2
12 K

Figure S4: **Coverage and top tool predictions of example DCS regions**

Coverage of one replicate of sub-sampled reads for sample 1 (row 1) and sample 2 (row 2) with the respective reference regions (row 3) in two example regions. The color of row 3 indicates up-regulation in sample 1 (red), up regulation in sample 2 (blue) and no difference (gray). The height of predicted regions represents the -log10 of p-value, adjusted p-value or FDR or the score derived from the respective DCS tool. Therefore, higher bars mean the respective DCS tool has more confidence in that region to be differential between the samples. **a,** Rows 4 to 11 show the predicted regions from all applied parameter setups of edgeR within this replicate of TF sub-sampled peaks (peak callers SICER2 and JAMM did not return peak-regions for this example region). **b**, Rows 4 to 9 show the predicted regions from all tested parameter setups of EpiCenter within this replicate of broad mark sub-sampled peaks (setup 5 did not result in predictions for the region shown, details of parameter setups: Table S3). **c**, Rows 4 to 8 show predicted regions from the best parameter setups of the top 5 DCS tools for TF and **d**, broad mark data and 50:50 regulation. Representative regions for sub-sampled (**a**, **c**) C/EBPa (TF) and (**b**, **d**) H3K36me3 (broad mark) in 50:50 regulation scenarios are shown.

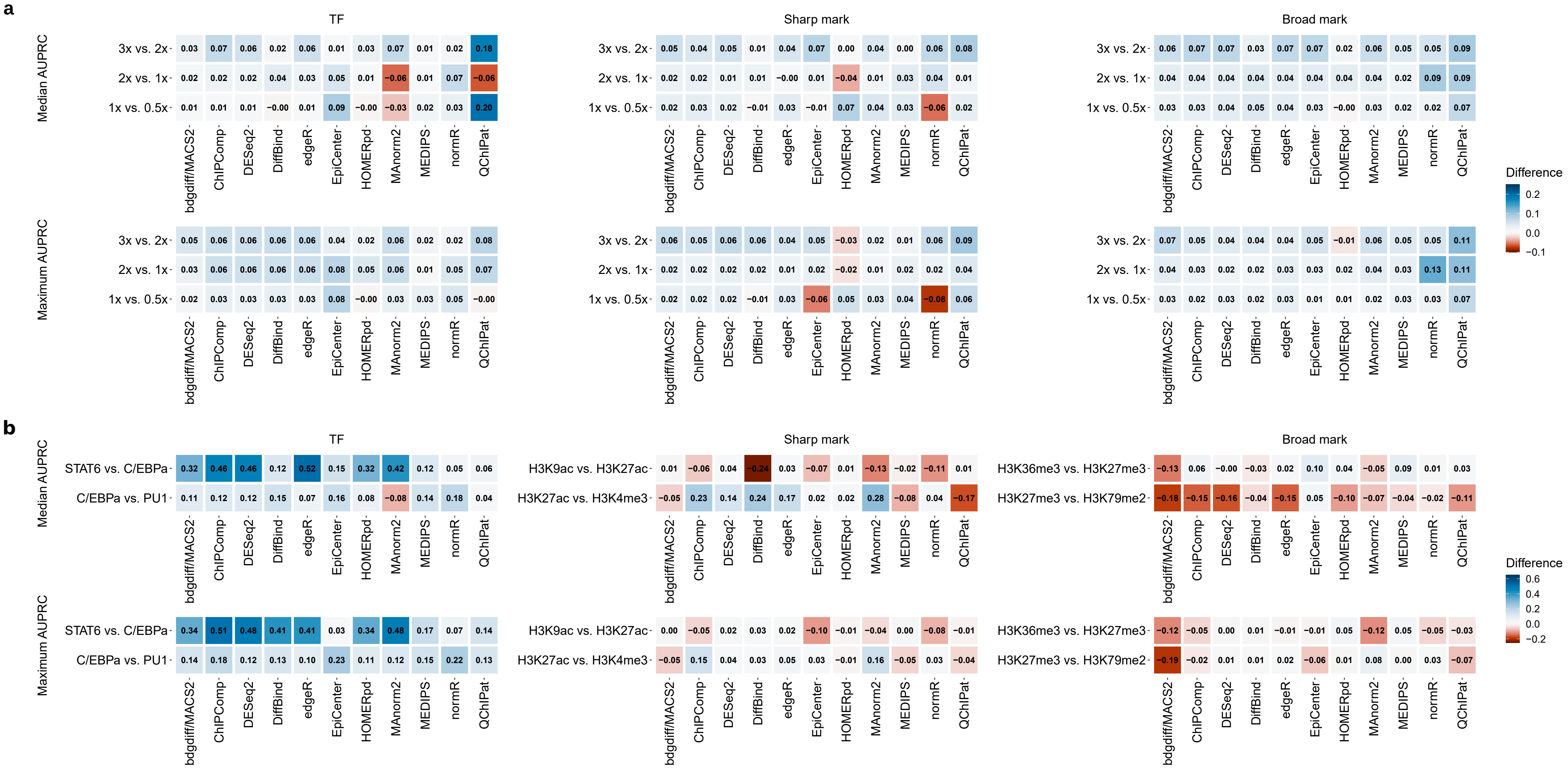
a
TF
Sharp mark
Broad mark
Median AUPRC
Maximum AUPRC
bdgdiff/MACS2 ChIPComp DESeq2 DiffBind edgeR EpiCenter HOMERpd MAnorm2 MEDIPS normR QChIPat
TF Median AUPRC 3x vs. 2x: 0.03 0.07 0.06 0.02 0.06 0.01 0.03 0.07 0.01 0.02 0.18
TF Median AUPRC 2x vs. 1x: 0.02 0.02 0.02 0.04 0.03 0.05 0.01 −0.06 0.01 0.07 −0.06
TF Median AUPRC 1x vs. 0.5x: 0.01 0.01 0.01 −0.00 0.01 0.09 −0.00 −0.03 0.02 0.03 0.20
Sharp mark Median AUPRC 3x vs. 2x: 0.05 0.04 0.05 0.01 0.04 0.07 0.00 0.04 0.00 0.06 0.08
Sharp mark Median AUPRC 2x vs. 1x: 0.02 0.02 0.01 0.01 −0.00 0.01 −0.04 0.01 0.03 0.04 0.01
Sharp mark Median AUPRC 1x vs. 0.5x: 0.02 0.03 0.02 −0.01 0.03 −0.01 0.07 0.04 0.03 −0.06 0.02
Broad mark Median AUPRC 3x vs. 2x: 0.06 0.07 0.07 0.03 0.07 0.07 0.02 0.06 0.05 0.05 0.09
Broad mark Median AUPRC 2x vs. 1x: 0.04 0.04 0.04 0.04 0.04 0.04 0.04 0.04 0.02 0.09 0.09
Broad mark Median AUPRC 1x vs. 0.5x: 0.03 0.03 0.04 0.05 0.04 0.03 −0.00 0.03 0.02 0.02 0.07
TF Maximum AUPRC 3x vs. 2x: 0.05 0.06 0.06 0.06 0.06 0.04 0.02 0.06 0.02 0.02 0.08
TF Maximum AUPRC 2x vs. 1x: 0.03 0.06 0.06 0.06 0.06 0.08 0.05 0.06 0.01 0.05 0.07
TF Maximum AUPRC 1x vs. 0.5x: 0.02 0.03 0.03 0.03 0.03 0.08 −0.00 0.03 0.03 0.05 −0.00
Sharp mark Maximum AUPRC 3x vs. 2x: 0.06 0.05 0.06 0.06 0.04 0.05 −0.03 0.02 0.01 0.06 0.09
Sharp mark Maximum AUPRC 2x vs. 1x: 0.02 0.02 0.02 0.02 0.01 0.02 −0.02 0.01 0.02 0.02 0.04
Sharp mark Maximum AUPRC 1x vs. 0.5x: 0.02 0.02 0.02 −0.01 0.03 −0.06 0.05 0.03 0.04 −0.08 0.06
Broad mark Maximum AUPRC 3x vs. 2x: 0.07 0.05 0.04 0.04 0.04 0.05 −0.01 0.06 0.05 0.05 0.11
Broad mark Maximum AUPRC 2x vs. 1x: 0.04 0.03 0.02 0.02 0.03 0.03 0.02 0.04 0.03 0.13 0.11
Broad mark Maximum AUPRC 1x vs. 0.5x: 0.03 0.02 0.03 0.02 0.03 0.01 0.01 0.02 0.03 0.03 0.07
Difference
0.2
0.1
0.0
−0.1
b
TF Median AUPRC STAT6 vs. C/EBPa: 0.32 0.46 0.46 0.12 0.52 0.15 0.32 0.42 0.12 0.05 0.06
TF Median AUPRC C/EBPa vs. PU1: 0.11 0.12 0.12 0.15 0.07 0.16 0.08 −0.08 0.14 0.18 0.04
Sharp mark Median AUPRC H3K9ac vs. H3K27ac: 0.01 −0.06 0.04 −0.24 0.03 −0.07 0.01 −0.13 −0.02 −0.11 0.01
Sharp mark Median AUPRC H3K27ac vs. H3K4me3: −0.05 0.23 0.14 0.24 0.17 0.02 0.02 0.28 −0.08 0.04 −0.17
Broad mark Median AUPRC H3K36me3 vs. H3K27me3: −0.13 0.06 −0.00 −0.03 0.02 0.10 0.04 −0.05 0.09 0.01 0.03
Broad mark Median AUPRC H3K27me3 vs. H3K79me2: −0.18 −0.15 −0.16 −0.04 −0.15 0.05 −0.10 −0.07 −0.04 −0.02 −0.11
TF Maximum AUPRC STAT6 vs. C/EBPa: 0.34 0.51 0.48 0.41 0.41 0.03 0.34 0.48 0.17 0.07 0.14
TF Maximum AUPRC C/EBPa vs. PU1: 0.14 0.18 0.12 0.13 0.10 0.23 0.11 0.12 0.15 0.22 0.13
Sharp mark Maximum AUPRC H3K9ac vs. H3K27ac: 0.00 −0.05 0.02 0.03 0.02 −0.10 −0.01 −0.04 0.00 −0.08 −0.01
Sharp mark Maximum AUPRC H3K27ac vs. H3K4me3: −0.05 0.15 0.04 0.03 0.05 0.03 −0.01 0.16 −0.05 0.03 −0.04
Broad mark Maximum AUPRC H3K36me3 vs. H3K27me3: −0.12 −0.05 0.00 0.01 −0.01 −0.01 0.05 −0.12 0.05 −0.05 −0.03
Broad mark Maximum AUPRC H3K27me3 vs. H3K79me2: −0.19 −0.02 0.01 0.01 0.02 −0.06 0.01 0.08 0.00 0.03 −0.07
Difference
0.6
0.4
0.2
0.0
−0.2

Figure S5: **Signal-to-noise ratio affects AUPRC**

Heatmaps showing the difference of AUPRC between lower and higher FRiP and higher and lower background signal for the top 11 DCS tools. **a**, The difference in AUPRC for simulated data with decreasing background, 3x versus 2x background, 2x versus 1x background and 1x versus 0.5x background. The upper row shows the differences in the median AUPRC and the lower row the differences in the maximum AUPRC per DCS tool. The left column shows the differences for TFs, middle column for sharp marks and the right column for broad marks. Colors indicate higher AUPRC (blue) for the sample with lower background, lower AUPRC (red) for the sample with lower background and now difference (white). Rounded values are shown inside tiles.

b, Heatmaps show differences in AUPRC for the top 11 DCS tools. The analyzed data is derived from sub-sampled ChIP-seq experiments from additional TFs and histone marks with different FRiP. Colors according to the differences in AUPRC between the samples with lowest vs. intermediate and intermediate vs. highest FRiP (lower AUPRC in lower FRiP sample: red, higher AUPRC in the higher FRiP sample: blue, no difference: white). The left column shows the differences for TFs, middle column for sharp marks and the right column for broad marks. The upper row shows the differences in the median AUPRC and the lower row the differences in the maximum AUPRC per DCS tool.

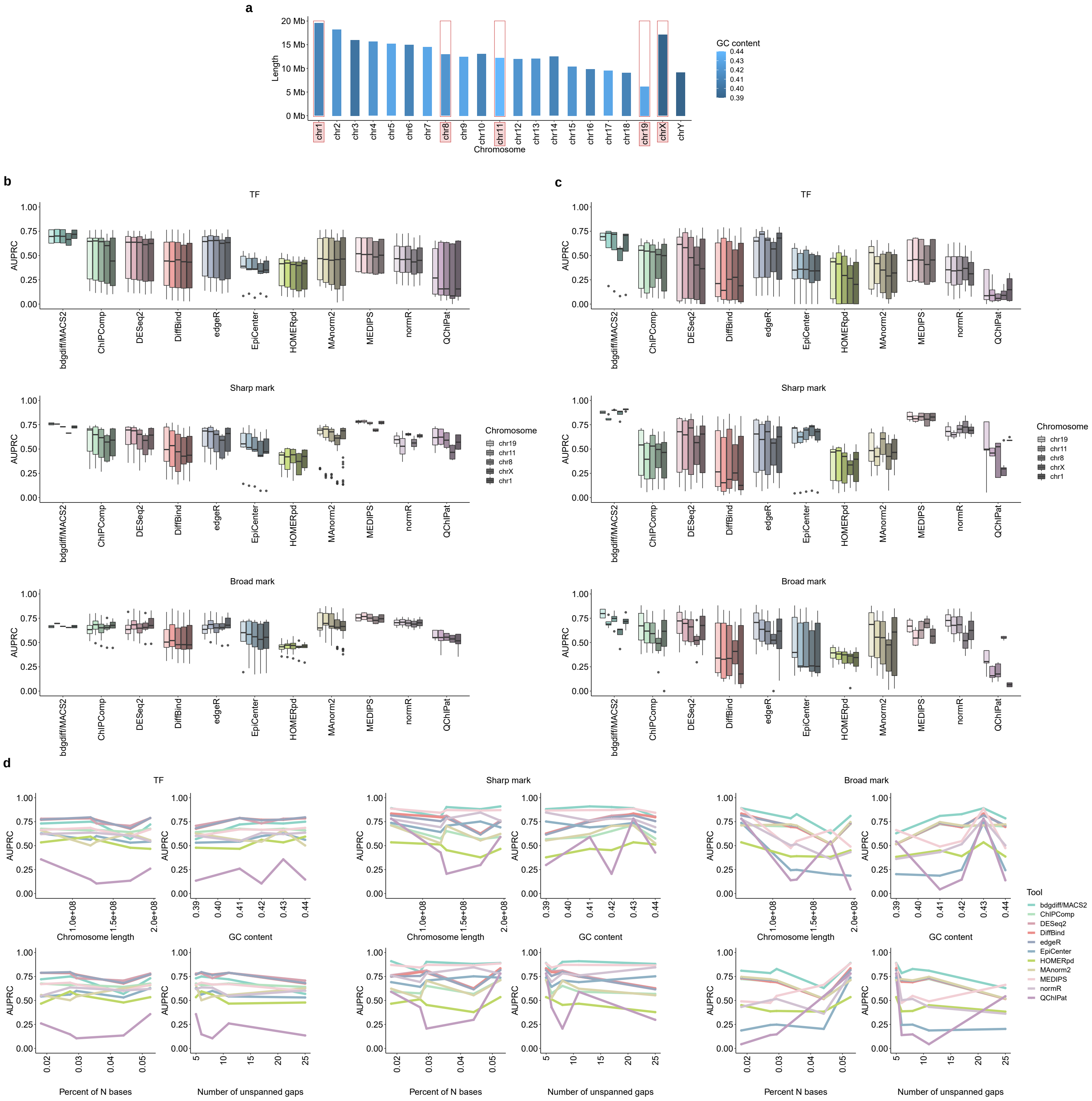

a
Length
20 Mb
15 Mb
10 Mb
5 Mb
0 Mb
chr1
chr2
chr3
chr4
chr5
chr6
chr7
chr8
chr9
chr10
chr11
chr12
chr13
chr14
chr15
chr16
chr17
chr18
chr19
chrX
chrY
Chromosome
GC content
0.44
0.43
0.42
0.41
0.40
0.39
b
c
TF
Sharp mark
Broad mark
AUPRC
bdgdiff/MACS2
ChIPComp
DESeq2
DiffBind
edgeR
EpiCenter
HOMERpd
MAnorm2
MEDIPS
normR
QChIPat
Chromosome
chr19
chr11
chr8
chrX
chr1
d
Chromosome length
GC content
Percent of N bases
Number of unspanned gaps
Tool
bdgdiff/MACS2
ChIPComp
DESeq2
DiffBind
edgeR
EpiCenter
HOMERpd
MAnorm2
MEDIPS
normR
QChIPat

Figure S6: **Effects of chromosome characteristics and ChIP-seq signal distribution on AUPRC**

a, GC content and chromosome length in the mouse genome assembly (mm10). The investigated chromosomes are highlighted in red. **b**, AUPRCs from simulated data of the top 11 DCS tools (based on AUPRC for the initial six shape and regulation scenarios) for five chromosomes of mm10 (chr1, chr8, chr11, chr19 and chrX) for TFs, sharp and broad marks. **c**, AUPRCs from sub-sampled data of the top 11 DCS tools for C/EBPa (TF), H3K27ac (sharp mark) and H3K36me3 (broad mark) data. Chromosomes per DCS tool are ordered by length, from short to long. Box plot limits, 25% and 75% quantiles; center line, median; whiskers, 1.5x interquartile range. **d**, Line graphs show the AUPRC for the best performing parameter setup per DCS tool (for the top 11 tools from the initial analysis based on chr19) for chromosome length, GC content, percent of unknown (N) bases and the number of unspanned gaps of the five chromosomes from the mm10 assembly.

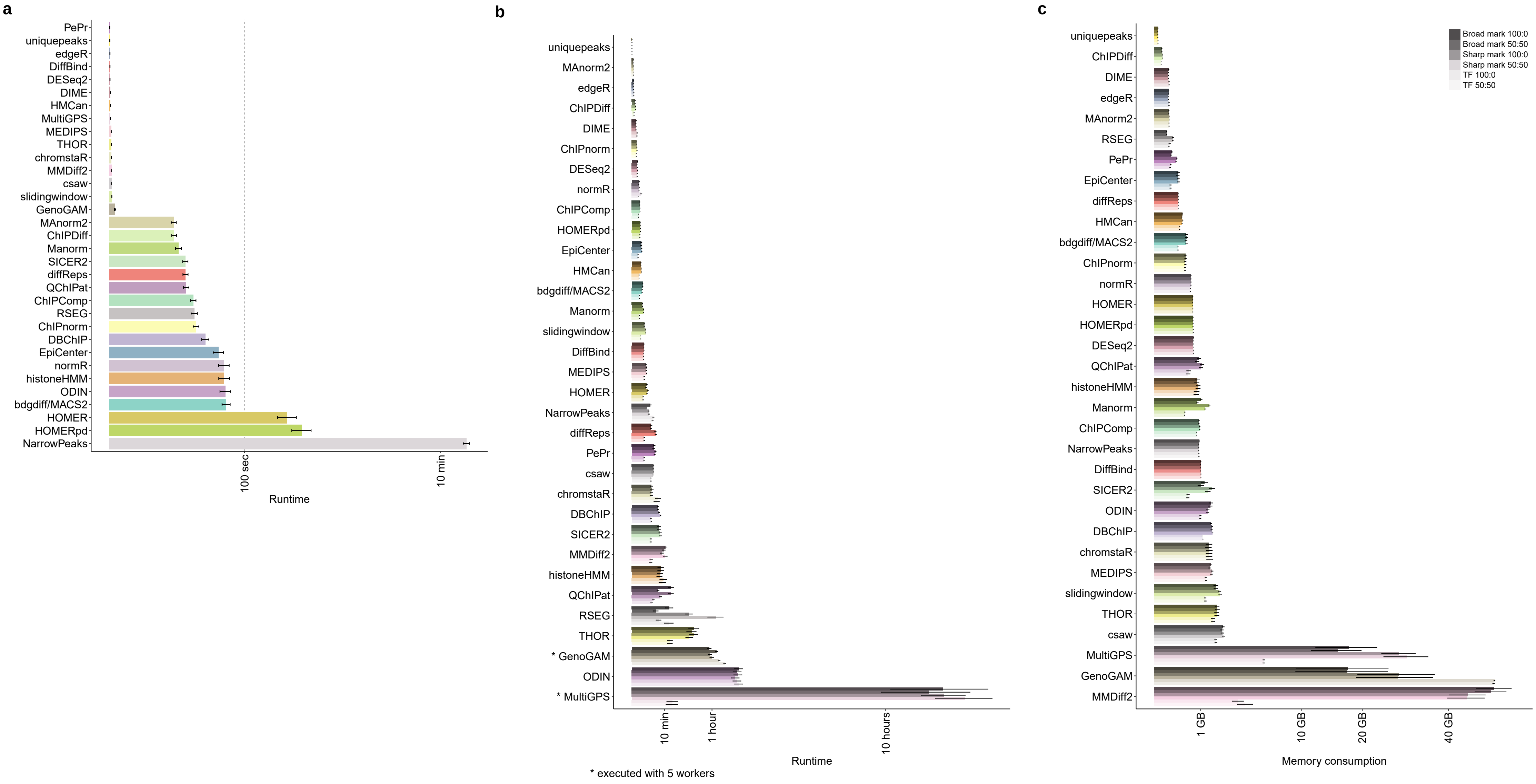
a
PePr
uniquepeaks
edgeR
DiffBind
DESeq2
DIME
HMCan
MultiGPS
MEDIPS
THOR
chromstaR
MMDiff2
csaw
slidingwindow
GenoGAM
MAnorm2
ChIPDiff
Manorm
SICER2
diffReps
QChIPat
ChIPComp
RSEG
ChIPnorm
DBChIP
EpiCenter
normR
histoneHMM
ODIN
bdgdiff/MACS2
HOMER
HOMERpd
NarrowPeaks
100 sec
10 min
Runtime
b
uniquepeaks
MAnorm2
edgeR
ChIPDiff
DIME
ChIPnorm
DESeq2
normR
ChIPComp
HOMERpd
EpiCenter
HMCan
bdgdiff/MACS2
Manorm
slidingwindow
DiffBind
MEDIPS
HOMER
NarrowPeaks
diffReps
PePr
csaw
chromstaR
DBChIP
SICER2
MMDiff2
histoneHMM
QChIPat
RSEG
THOR
* GenoGAM
ODIN
* MultiGPS
10 min
1 hour
10 hours
Runtime
* executed with 5 workers
c
uniquepeaks
ChIPDiff
DIME
edgeR
MAnorm2
RSEG
PePr
EpiCenter
diffReps
HMCan
bdgdiff/MACS2
ChIPnorm
normR
HOMER
HOMERpd
DESeq2
QChIPat
histoneHMM
Manorm
ChIPComp
NarrowPeaks
DiffBind
SICER2
ODIN
DBChIP
chromstaR
MEDIPS
slidingwindow
THOR
csaw
MultiGPS
GenoGAM
MMDiff2
1 GB
10 GB
20 GB
40 GB
Memory consumption
Broad mark 100:0
Broad mark 50:50
Sharp mark 100:0
Sharp mark 50:50
TF 100:0
TF 50:50

Figure S7: **Preparation time and runtime plus memory requirements based on individual scenarios**
a, Average preparation time to reformat and process input files depending on the respective DCS tool over the six test scenarios. **b**, Runtime and **c**, memory consumption of all tested DCS tools per peak and regulation scenario. Due to their extensive runtimes GenoGAM and MultiGPS where executed with 5 workers. Whiskers indicate standard error of the mean.

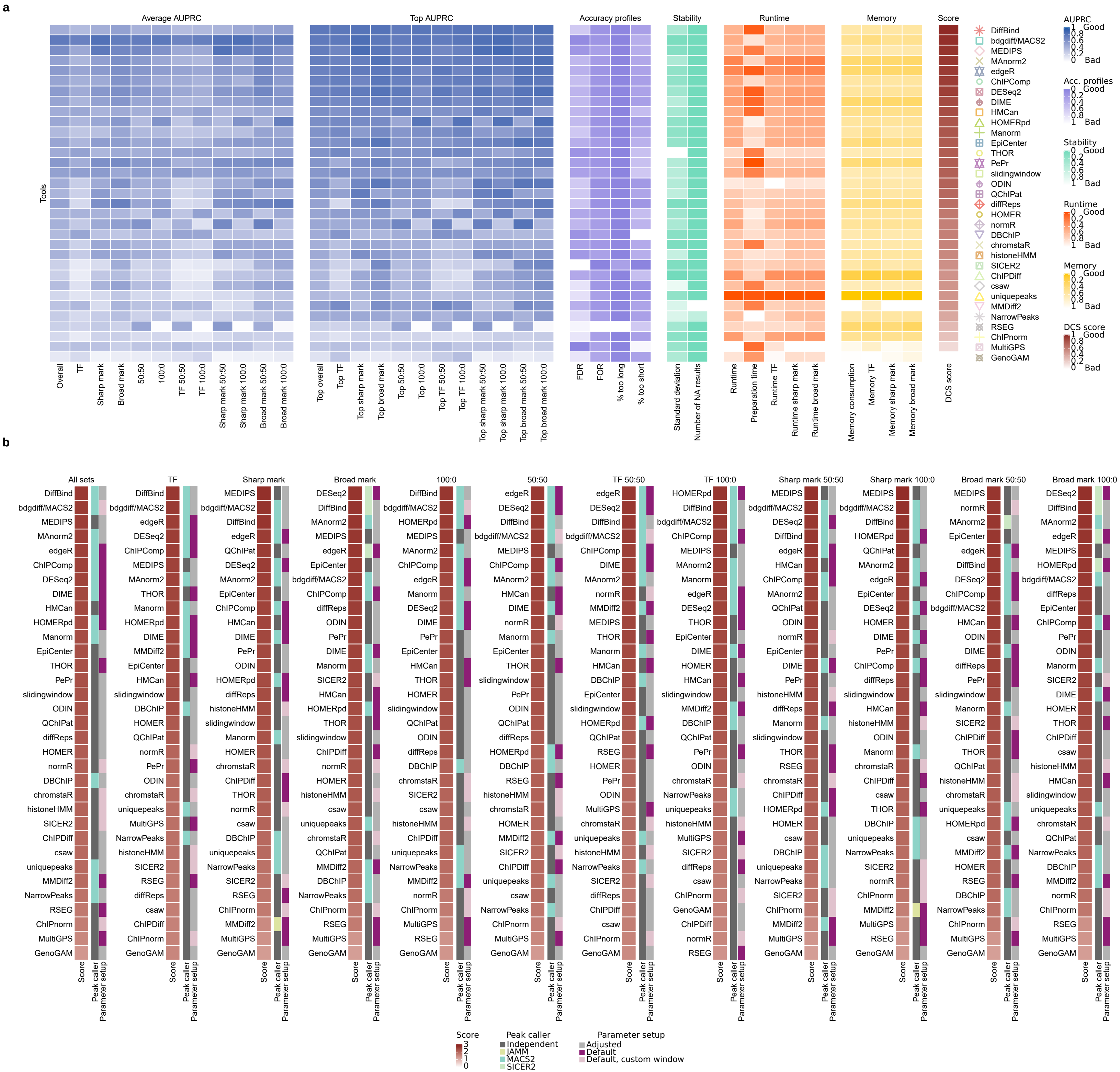
a
Average AUPRC
Overall, TF, Sharp mark, Broad mark, 50:50, 100:0, TF 50:50, TF 100:0, Sharp mark 50:50, Sharp mark 100:0, Broad mark 50:50, Broad mark 100:0
Top AUPRC
Top overall, Top TF, Top sharp mark, Top broad mark, Top 50:50, Top 100:0, Top TF 50:50, Top TF 100:0, Top sharp mark 50:50, Top sharp mark 100:0, Top broad mark 50:50, Top broad mark 100:0
Accuracy profiles
FDR, FOR, % too long, % too short
Stability
Standard deviation, Number of NA results
Runtime
Runtime, Preparation time, Runtime TF, Runtime sharp mark, Runtime broad mark
Memory
Memory consumption, Memory TF, Memory sharp mark, Memory broad mark
Score
DCS score
Tools
DiffBind, bdgdiff/MACS2, MEDIPS, MAnorm2, edgeR, ChIPComp, DESeq2, DIME, HMCan, HOMERpd, Manorm, EpiCenter, THOR, PePr, slidingwindow, ODIN, QChIPat, diffReps, HOMER, normR, DBChIP, chromstaR, histoneHMM, SICER2, ChIPDiff, csaw, uniquepeaks, MMDiff2, NarrowPeaks, RSEG, ChIPnorm, MultiGPS, GenoGAM
AUPRC: 1 0.8 0.6 0.4 0.2 0; Good, Bad
Acc. profiles: 0 0.2 0.4 0.6 0.8 1; Good, Bad
Stability: 0 0.2 0.4 0.6 0.8 1; Good, Bad
Runtime: 0 0.2 0.4 0.6 0.8 1; Good, Bad
Memory: 0 0.2 0.4 0.6 0.8 1; Good, Bad
DCS score: 1 0.8 0.6 0.4 0.2 0; Good, Bad
b
All sets: DiffBind, bdgdiff/MACS2, MEDIPS, MAnorm2, edgeR, ChIPComp, DESeq2, DIME, HMCan, HOMERpd, Manorm, EpiCenter, THOR, PePr, slidingwindow, ODIN, QChIPat, diffReps, HOMER, normR, DBChIP, chromstaR, histoneHMM, SICER2, ChIPDiff, csaw, uniquepeaks, MMDiff2, NarrowPeaks, RSEG, ChIPnorm, MultiGPS, GenoGAM
TF: DiffBind, bdgdiff/MACS2, edgeR, DESeq2, ChIPComp, MEDIPS, MAnorm2, THOR, Manorm, HOMERpd, DIME, MMDiff2, EpiCenter, HMCan, slidingwindow, DBChIP, HOMER, QChIPat, normR, PePr, ODIN, chromstaR, uniquepeaks, MultiGPS, NarrowPeaks, histoneHMM, SICER2, RSEG, diffReps, csaw, ChIPDiff, ChIPnorm, GenoGAM
Sharp mark: MEDIPS, bdgdiff/MACS2, DiffBind, edgeR, QChIPat, DESeq2, MAnorm2, EpiCenter, ChIPComp, HMCan, DIME, PePr, ODIN, HOMERpd, diffReps, histoneHMM, slidingwindow, Manorm, HOMER, chromstaR, ChIPDiff, THOR, normR, csaw, DBChIP, uniquepeaks, NarrowPeaks, SICER2, RSEG, ChIPnorm, MMDiff2, MultiGPS, GenoGAM
Broad mark: DESeq2, DiffBind, MAnorm2, MEDIPS, edgeR, EpiCenter, bdgdiff/MACS2, ChIPComp, diffReps, ODIN, PePr, DIME, Manorm, SICER2, HMCan, HOMERpd, THOR, slidingwindow, ChIPDiff, normR, HOMER, histoneHMM, csaw, uniquepeaks, chromstaR, QChIPat, MMDiff2, DBChIP, NarrowPeaks, ChIPnorm, RSEG, MultiGPS, GenoGAM
100:0: DiffBind, bdgdiff/MACS2, HOMERpd, MEDIPS, MAnorm2, ChIPComp, edgeR, Manorm, DESeq2, DIME, PePr, EpiCenter, HMCan, THOR, HOMER, slidingwindow, QChIPat, ODIN, diffReps, DBChIP, chromstaR, SICER2, csaw, histoneHMM, ChIPDiff, uniquepeaks, NarrowPeaks, MMDiff2, normR, ChIPnorm, MultiGPS, RSEG, GenoGAM
50:50: edgeR, DESeq2, DiffBind, bdgdiff/MACS2, MEDIPS, ChIPComp, MAnorm2, HMCan, DIME, normR, Manorm, EpiCenter, THOR, slidingwindow, PePr, ODIN, QChIPat, diffReps, HOMERpd, DBChIP, RSEG, histoneHMM, chromstaR, HOMER, MMDiff2, SICER2, ChIPDiff, uniquepeaks, csaw, NarrowPeaks, ChIPnorm, MultiGPS, GenoGAM
TF 50:50: edgeR, DESeq2, DiffBind, bdgdiff/MACS2, ChIPComp, DIME, MAnorm2, normR, MMDiff2, MEDIPS, THOR, Manorm, HMCan, DBChIP, EpiCenter, slidingwindow, HOMERpd, QChIPat, RSEG, HOMER, PePr, ODIN, MultiGPS, chromstaR, uniquepeaks, histoneHMM, NarrowPeaks, SICER2, diffReps, ChIPDiff, csaw, ChIPnorm, GenoGAM
TF 100:0: HOMERpd, DiffBind, bdgdiff/MACS2, ChIPComp, MEDIPS, MAnorm2, Manorm, edgeR, DESeq2, THOR, EpiCenter, DIME, HOMER, HMCan, slidingwindow, MMDiff2, DBChIP, QChIPat, PePr, ODIN, chromstaR, NarrowPeaks, uniquepeaks, histoneHMM, MultiGPS, SICER2, diffReps, csaw, ChIPnorm, GenoGAM, ChIPDiff, normR, RSEG
Sharp mark 50:50: MEDIPS, bdgdiff/MACS2, DESeq2, DiffBind, edgeR, HMCan, ChIPComp, MAnorm2, QChIPat, ODIN, normR, EpiCenter, DIME, PePr, histoneHMM, diffReps, Manorm, slidingwindow, THOR, RSEG, chromstaR, ChIPDiff, HOMERpd, HOMER, csaw, DBChIP, uniquepeaks, NarrowPeaks, SICER2, ChIPnorm, MMDiff2, MultiGPS, GenoGAM
Sharp mark 100:0: MEDIPS, bdgdiff/MACS2, DiffBind, HOMERpd, QChIPat, MAnorm2, edgeR, EpiCenter, DESeq2, HOMER, DIME, PePr, ChIPComp, diffReps, slidingwindow, HMCan, histoneHMM, ODIN, Manorm, chromstaR, ChIPDiff, csaw, THOR, DBChIP, uniquepeaks, NarrowPeaks, SICER2, normR, ChIPnorm, MMDiff2, MultiGPS, RSEG, GenoGAM
Broad mark 50:50: MEDIPS, normR, MAnorm2, EpiCenter, edgeR, DiffBind, DESeq2, ChIPComp, bdgdiff/MACS2, HMCan, ODIN, DIME, diffReps, PePr, slidingwindow, Manorm, SICER2, ChIPDiff, THOR, QChIPat, histoneHMM, chromstaR, uniquepeaks, HOMERpd, csaw, MMDiff2, HOMER, RSEG, DBChIP, NarrowPeaks, ChIPnorm, MultiGPS, GenoGAM
Broad mark 100:0: DESeq2, DiffBind, MAnorm2, edgeR, MEDIPS, HOMERpd, bdgdiff/MACS2, diffReps, EpiCenter, ChIPComp, PePr, ODIN, Manorm, SICER2, DIME, HOMER, THOR, ChIPDiff, csaw, histoneHMM, HMCan, slidingwindow, uniquepeaks, chromstaR, QChIPat, NarrowPeaks, DBChIP, MMDiff2, normR, ChIPnorm, RSEG, MultiGPS, GenoGAM
Score, Peak caller, Parameter setup
Score: 3 2 1 0
Peak caller: Independent, JAMM, MACS2, SICER2
Parameter setup: Adjusted, Default, Default, custom window

Figure S8: **Performance measures and ranking of DCS tools per scenario**

a, Heatmap of DCS tool performance. The first two columns represent the average AUPRCs of all parameter setups per DCS tool and the top AUPRCs the best performing parameter setup per DCS tool for the six tested peak shape and regulation scenarios and their combinations. All other metrics are shown as average of all parameter setups per DCS tool over all test scenarios. In columns 3 and 4 the accuracy profiles and the stability in form of standard deviations between AUPRCs of the simulated and sub-sampled replicates and the number of NA results summarizing all failed and faulty execution runs or runs with empty outputs are shown. The runtime columns consist of the preparation time, time required for the DCS prediction on average and the runtime per peak-shape as the number of reads to process differ. The last columns show memory consumption per DCS tool over all test sets and for TFs, sharp and broad marks only. Tools were ordered by their mean DCS score over all test sets. **b**, The best performing parameter setups per DCS tool were ranked by DCS score for the respective scenario and scenario combinations (for details see Tables S2, S3, S4 and S5). Colored boxes indicate the applied peak caller if applicable and if default, default with custom windows or adjusted parameters were used.
