# Supplementary material for: Comprehensive assessment of differential ChIP-seq tools guides optimal algorithm selection
Source: Genome Biol. 2022 May 24;23:119. doi: 10.1186/s13059-022-02686-y (PMC9128273; doi:10.1186/s13059-022-02686-y)
Supplement: Supplementary file 7 — Additional file 7. Review history. [file 13059_2022_2686_MOESM7_ESM.docx]

**1^st^ round**

**Reviewer 1**

The authors present an evaluation of 31 computational tools for differential ChIP-seq peak analysis. To this end, they perform evaluations using both synthetic and sub-sampled ChIP-seq differential datasets. Biologically motivated choices are used throughout, which is a major plus (e.g., TF-type peaks are represented by CEBP data, K27ac is used for "sharp" peaks, and K36me3 for "broad" peaks). To my knowledge, an evaluation such as this has never before been performed on this scale, and I commend the authors on their overall thoroughness and comprehensive inclusion of many different methods.


Major issues

1. Confusion regarding the nature of the sub-sampled dataset

I am confused about the reference datasets used in the evaluations - are there 2 or 3? Is DCSsub creating one reference dataset and sub-sampling creating a different one? If so, what is the difference between them?

It is not clear what data are being used to create the sub-sampling datasets. The GEO accession numbers are for series, rather than individual data sets. Additional details should be included. Is it one ChIP-seq experiment that is sub-sampled, then put through a beta distribution to create differential peaks? Or is it sub-sampling of multiple conditions?

How are TPs/TNs identified for the "real" test datasets? I understand how this would be done for simulated data, but how do you know the "ground truth" e.g. on the CEBP subsampled peaks?

Overall, the sub-sampled evaluation set is not very well explained. Please provide clearer text (including motivation for why sub-sampling is needed, and explanation of how the sum-sampling was performed), along with a picture similar to Fig 1A showing how the sub-sampling works.


----------------------------------------------------------------------------

2. The AUPRC values are based on both the simulated and sub-sampled datasets. Does the ordering of the top results change if the AUPRC for just the sub-sampled data is used?

----------------------------------------------------------------------------

3. Peak shape is important, but so is signal to noise. It would have been nice to also evaluate performance at a few different FRIP ranges. I would think that some tools would be much more capable of handling noisier datasets (e.g., from experiments performed in primary cells) than others, and this is very important information when deciding which method to use.

----------------------------------------------------------------------------


===============================================

Minor issues

The text focuses on transcription factors and hardly mentions histone modifications, yet the histone modifications are an important focus of ChIP-seq experiments, and the comparative analysis they did. The text should be modified to reflect this.

Its hard to tell the difference in performance in the Fig6A heatmap. Instead of alphabetical sorting, can the rows be sorted by some sort of mean/median score across the performance metrics? This will make the figure much more useful for a lab that needs to pick one or two methods

Is it possible to at least somewhat quantify "ease of installation"? that is also a very important consideration. for example, "order of magnitude" amount of time between having the tool downloaded and getting it up and running (e.g., minutes, hours, days…) I realize this will depend on the individual or the particular environment (and hence, sometimes luck), but it would be useful to know even roughly how hard it is to install these tools, since of course this can vary substantially from one tool to the other

The last paragraph in the results section is long and seems to contain multiple topics - please split

DCS score seems very arbitrary: "For the final score we summed up the metrics using the following weights: AUPRC 1.6, standard deviation and number of NAs 0.2, runtime 0.4, preparation time 0.2, and memory consumption 0.4." - please provide some sort of intuitive justification

Lines 152-158: Highlighting the top five tool parameter setup combinations based on AUPRC values, for each regulation scenario and peak shape, showed that edgeR, DESeq2 [32] or bdgdiff/MACS2 were prominently represented among the top performing tools across all conditions (Figure 3c, top tools with exact parameter setups are shown in Additional file 4: Figure S3). MEDIPS performed very well in the analysis of sharp marks. HOMERpd [33] (peak dependent run) and DiffBind [34], both tools with previous peak calling via MACS2 or SICER2, yielded very good results for the 100:0 scenarios, irrespective of peak shape.
In Figure 3c, DiffBind is in the top 5 for each scenario, but the text implies the performance is good only in the 100:0 scenarios. Why is that?

Lines 161-164: As DCS tools rank their results based on various metrics, such as p-value, FDR or diverse scores that are not compatible with a uniform threshold, we focused on the top 300 predicted regions and set the threshold just below the lowest number of differential regions present in any test case.
This sentence doesn't make sense to me. What threshold?

Lines 175-177: The FDR was below 0.2 in all cases, and even lower for histone marks, while the FOR was lower than the FDR across all samples (Figure 4b).
Figure 4b shows that the FOR is not lower than the FDR for some of the broad mark samples. The text should be changed from "all samples" to "most samples"

Lines 184-186: In this case, SICER2 derived peaks were used for the analyses with HOMERpd, DiffBind and DESeq2, MACS2 peaks for MAnorm2, and MEDIPS is peak independent.
Why was a different peak caller used for MAnorm2?

Lines 231-233: The fraction of predicted regions that are shorter than the reference was between 0.002% and 0.31%. Here, the negative outliers were DBChip and MultiGPS with 0.31% and 0.29%, respectively.
I don't understand this wording - 0.31% and 0.29% aren't outliers.

Line 406: Duplicate single-end reads were kept.
Why? That's not standard practice.

Lines 417-422: Change the order of the sentences - describe SICER2, then MACS2

Line 434: HMCan is missing the reason why it was run on the other computer.

Lines 653-666 (Legend for Figure S4). The legends aren't associated with the correct panels. Panel "a" has the text for panel "c". Panel "b" has the text for panel "d". Panel "c" has the text for panel "a". Panel "d" has the text for panel "b". Additionally, line 659 should be "broad mark", not "sharp mark".

Include panel number when referring to supplementary figures (e.g., Figure S4a rather than just Figure S4).

Provide more details on the transcription factor and histone marks (some readers may not be familiar with them). e.g., H3K27ac is found on enhancers and promoters; H3K36me3 covers gene bodies; C/EBPa is involved in ...

Make sure all "Parameter setup" values in Table S3 are present in Table S2. For instance, the values for DiffBind in Table S3 don't match what is in Table S2.

Provide details on uniquepeaks - there's no citation or download link in Table S1.

What exactly does "off-the-shelf" mean? (Different people tend to use it slightly differently).

Figure 4b: The plots on the right-hand side should be the same size as the others.

Figure S6a: The random regions aren't included (see lines 449,451)

Table S1: The "Focus" column is really informative. Please indicate which tools have a focus on RNA-seq.

Lines 48-50: quantitative comparison of protein-DNA interactions between different biological scenarios requires a priori knowledge of genomic regions that are occupied by the protein under investigation.
Why? What types of knowledge? I don't quite follow the reasoning here.

Lines 56-58: For instance, some tools, particularly those initially developed for RNA-seq analysis, assume that the majority of occupied genomic regions do not differ between experimental states. Yet, this is often not the case in ChIP-seq datasets.
(Regarding the statement about the ChIP-seq datasets) Really? Is this common/general knowledge or is there a citation?

Lines 59-60: Furthermore, depending on the biological scenario, different normalization methods employed by individual tools will have a strong impact on the outcome.
Any examples/citations?

===============================================

Typos / grammatical errors

Line 61: ranging from few hundred to several thousands
It should be "ranging from a few hundred" (missing the "a")

Lines 74-75: In addition, our decision trees also provide recommendations for the analysis of ChIP-seq datasets of
Remove either "In addition" or "also" - including both is redundant

Line 114: evaluation pipeline (Figure 1b), which includes, alignment against the respective reference genomes
Remove the comma after the word includes

Lines 171-172: Furthermore, as a subset of DCS tools depends on predefined window sizes, these impacts on their predictions.
Change "predefined window sizes" to "a predefined window size"
Change "these impacts on their predictions" to "this impacts their predictions"

Lines 224-225: In particular, RSEG was not capable to successfully deal with a global down-regulation of ChIP-seq signals in one sample.
Change "capable" to "able"

Lines 234-236: There were no empty output files or failed execution runs for most tools, while only few runs with the MMDiff2 (14.6%) and NarrowPeaks (7.6%) tools failed.
Change "while only" to "except for a"
Change "failed" to "that failed"

Line 236: For MMDiff2 memory allocation failed for individual peak
Remove "For" at the beginning of the sentence.

Line 242: towards the optimal setup of DCS tool
It should be "towards the optimal setup of a DCS tool" (missing the "a")

Line 252: we provide decision trees that will help to guide biomedical scientists
Remove "to"

Line 289: investigators might chose the tools which showed
Change "chose" to "choose"

Line 293: we suggest to use the tools
Change "to use" to "using"

Line 317: were checked against a black list
Change "black list" to "blacklist"

Line 328: In addition, also noise was added to the samples and their replicates.
Remove "also"

Line 334: was stored in bed file
Change "was" to "were"

Lines 342-343: with repeat-masked regions greater 1kb as black list.
Change to "with repeat-masked regions greater than 1kb as a blacklist."

Line 397: Change "Hiseq2500" to "HiSeq 2500"

Line 459: and data were combined this with
Remove "this"

Line 470: The top 300 predicted peaks
Change to "The top 300 predicted differential peaks"

Line 473: The minimum of simulated
Change to "The minimum number of simulated"

Line 477: The FOR was also calculated by
Remove "also"

Line 525: used the following public available datasets
Change "public" to "publicly"

Line 625: from a H3K27ac histone mark
Change "a" to "an"

Line 627: from a H3K36me3 histone mark
Change "a" to "an"

**Reviewer 2**

The manuscript presents a comprehensive and detailed assessment of bioinformatics approaches for 'differential' analysis of ChIP-Seq data. I find the design of the pipeline to be very careful and reasonable. The conclusions are solid and practically useful and the visualization of the results is truly beautiful.
Yet, there are some important issues to be addressed.

Major
(1) As far as I understood from the Methods, the authors used a single mouse chromosome (chr19) to perform all analyses. Many top-scoring approaches were using macs2 peak calls, with macs2 being executed with 'default' settings.
According to the Methods and Supplementary Data, for some reason, the genome size was not specifically set for macs2 callpeaks (see the -g parameter of macs2).
It is strange, as chr19 size was explicitly specified for a competing approach JAMM (Table S2).
I am not sure how it affects the results of the peak calling in this particular setting, but it seems a major issue that could change the outcome of the downstream analysis.

(2) It is unclear why chr19 was selected for analysis. Chromosomes are very non-uniform in terms of nucleotide composition and ChIP-Seq signal distribution, so a genome-wide estimate might provide more stable results.
As it might be unfeasible due to runtime constraints, a subset of 3-5 chromosomes could be still much more representative.
I suggest replicating at least a part of the analysis using a wider set of chromosomes (maybe re-check the performance of a small set of top-performing tools).

(3) Only a single ChIP-Seq dataset of each 'peak shape type' was used for evaluation. It is unclear if the underlying data had low or high sequencing depth, and whether the QC metrics (e.g. those suggested by the ENCODE ChIP-Seq analysis guidelines) were fulfilled by the utilized data. This should be checked and explicitly stated in the manuscript. Similar to (2) it might be very useful to replicate at least part of the analysis on different ChIP-Seq data of the same type (TF, narrow, or broad marks), as its signal-to-noise ratio can be dramatically different depending on a cell type or antibody.

Minor
Page 2 - "all physiological processes" - I would suggest rephrasing this sentence. There is plenty of cellular machinery acting w/o DNA-protein interactions, and I would avoid talking about 'physiological' processes (with ChIP-Seq, we are on the molecular level).

Page 3 - "qualitative or quantitative" - I understand the intention of the authors to highlight differential analysis of ChIP-Seq data, but I consider all steps of the analysis as 'quantitative'. I suggest rephrasing this sentence.

Page 3 - "parameter-guided" what does it mean here?

Page 6 - "accuracy" - this word has a very particular technical meaning in machine learning, and I strongly suggest avoiding it when referencing a 'generic' predictive power of a model. Please consider 'performance measures', 'prediction quality' etc as possible alternatives.

Page 7 - Here it is unclear what are "accuracy profiles" and "differentially represented" genomic regions.

Page 7 - "top 300 predicted regions" - does it mean the predictions were evaluated at the fixed positive prediction rate? That is OK, but the end of the sentence is confusing "lowest number of differential regions present in any test case". What does it mean?

Page 8 - "TF peaks were not accurately detected with edgeR" - edgeR does not perform peak calling.

Page 8 - EpiCenter used with a narrow window for broad peaks. First, why? Second, this is more suited for Discussion.

Page 13 - "protein clusters". I find this terminology rather confusing. It might be better to stick to "clusters of binding sites/regions".

Page 15 - "background noise was set ..." Methods lack details regarding background noise simulation. 

Page 17 - usage of spike-ins requires a bit more details. Which particular tools were provided with external scaling factors? 

Figure 1c requires Y-axis label. Overall, the Figures clarity and style are top-notch, but figure captions are extremely brief. I would suggest providing more details.

**Authors’ response**

We want to thank both reviewers for their comments, their helpful and constructive feedback and the effort they put into this review. We highly appreciate the detailed and thorough analysis of our manuscript and we believe that it will strongly improve the quality and relevance of our work.

We are certain that the suggested changes help to better communicate our findings to the readers of Genome Biology. We hope that the current, extensively revised manuscript will meet the expectations of the editorial board and the reviewers of Genome Biology and merit publication of our manuscript in this journal.

The revised version of the manuscript contains two additional main figures that illustrate the influence of background noise, FRIP, chromosome size and nucleotide composition on tool performance. In addition, the revised manuscript contains two novel supplementary figures and two novel tables.

We addressed all points in detail. In sum, we included 48 new test sets (21 for the FRIP analysis and 27 for the analysis of additional chromosomes), which took almost two months to be processed on our hardware.

During the work on the revision, we identified a suboptimal assumption regarding failed DCS runs and tools that finished without output, which had a minor impact on their overall AUPRC in certain situations. This almost exclusively affected DCS tools with low performance and led to a slightly different order of tool performance when AUPRCs were used as a ranking metric. We corrected this minor error and updated Figures: 2, 3, S3, 4, 8 (former 6) and S8 (former S6). A point-by-point reply to the remarks of the reviewers is provided below.

The requested changes to the text are highlighted in yellow in the manuscript file.

Reviewer #1

The authors present an evaluation of 31 computational tools for differential ChIP-seq peak analysis. To this end, they perform evaluations using both synthetic and sub- sampled ChIP-seq differential datasets. Biologically motivated choices are used throughout, which is a major plus (e.g., TF-type peaks are represented by CEBP data, K27ac is used for "sharp" peaks, and K36me3 for "broad" peaks). To my knowledge, an evaluation such as this has never before been performed on this scale, and I commend the authors on their overall thoroughness and comprehensive inclusion of many different methods.

Major issues

1. Confusion regarding the nature of the sub-sampled dataset Comment: I am confused about the reference datasets used in the evaluations - are there 2 or 3? Is DCSsub creating one reference dataset and sub-sampling creating a different one? If so, what is the difference between them?

Response: We created two datasets for each peak shape/regulation setup, one from DCSsim and one from DCSsub. DCSsub does the sub-sampling from genuine ChIP- seq data. We used the term sub-sampling to refer to data generated by DCSsub. This should not be confused with random sub-sampling, which would not result in a reference of regulated peaks/regions required for the evaluation. We clarified the respective section in the manuscript (page 5 lines 106-108, page 7 lines 146-151).

Comment: It is not clear what data are being used to create the sub-sampling datasets. The GEO accession numbers are for series, rather than individual data sets. Additional details should be included. Is it one ChIP-seq experiment that is sub-sampled, then put through a beta distribution to create differential peaks? Or is it sub-sampling of multiple conditions?

Response: We included additional information about the ChIP-seq datasets that were used for sub-sampling in the “Methods” (page 20, lines 473-480) and “Availability of data and materials” (page 28 lines 671-680) sections. We also included Figure S1c to illustrate the sub-sampling approach and extended the description of how DCSsub was applied in the methods section (page 21 lines 492-509).

Comment: How are TPs/TNs identified for the "real" test datasets? I understand how this would be done for simulated data, but how do you know the "ground truth" e.g. on the CEBP subsampled peaks?

Response: The user defines TPs as part of the input parameters for DCSsub. In our experiments, we selected the top ~1000 peaks (by score, p-value or FDR) and provided them as a bed file. TNs represent everything else that is left in the selected chromosome. The aligned reads of peaks that are not covered by the bed file featuring the top peaks were randomly distributed between samples. This approach yielded peaks that are called over to the respective input control, but they are not differentially regulated between the two samples generated by DCSsub (pages 20-22, lines 473- 515).

Comment: Overall, the sub-sampled evaluation set is not very well explained. Please provide clearer text (including motivation for why sub-sampling is needed, and explanation of how the sum-sampling was performed), along with a picture similar to Fig 1A showing how the sub-sampling works.

Response: We added the sub-sampling data to acquire a more realistic signal-to-noise ratio and more fluctuation between regions to better model the “real-life” situation with ChIP-seq data. We extended the respective text sections in the background (page 5, lines 106-112) and results sections of the manuscript (pages 6-7, lines 141-151). We added sub-panel c in Figure S1 to illustrate how DCSsub works. We extended the section describing how DCSsub works (pages 20-21, lines 473-491) and the section on how DCSsub was applied in the methods section (pages 21-22, lines 492-533).

Comment: 2. The AUPRC values are based on both the simulated and sub-sampled datasets. Does the ordering of the top results change if the AUPRC for just the sub- sampled data is used?

Response: As simulated and sub-sampled data can have different advantages and disadvantages, we combined both approaches in an effort to overcome or at least reduce their individual disadvantages. The text in the results section was modified accordingly (pages 6-7, lines 141-151).

To answer the question: Yes, if the ordering of the top tools was based on the AUPRC of subsampled data only, it would be slightly different in comparison to the order based on the merged AUPRC. However, the specific rank of a DCS tool can be misleading if sets of tools with very similar AUPRCs are compared. For example, in the case of tool performance in evaluating broad marks with 100:0 regulation, a change in the 3rd or 4th decimal place of the AUPRC value would result in a different rank. Thus, exclusive inspection of ranked tools might suggest that a big difference exists between them, while they all might show very similar performance in reality. To show the influence of the “data source” on the AUPRC we added figure S2a, in which we show the differences between AUPRCs from simulated and subsampled data and the merged AUPRCs for the best performing tools per test scenario (page 6-7, lines 141-151).

Comment: 3. Peak shape is important, but so is signal to noise. It would have been nice to also evaluate performance at a few different FRIP ranges. I would think that some tools would be much more capable of handling noisier datasets (e.g., from experiments performed in primary cells) than others, and this is very important information when deciding which method to use.

Response: We fully agree that the signal-to-noise ratio of a given ChIP-seq experiment is highly relevant. To investigate its influence in tool performance, we simulated additional data sets and compared their AUPRC. As it is not possible to directly influence the distribution of reads into peak- and non-peak regions with DCSsim we chose to increase and decrease the background signal of the newly simulated data. We tested 0.5x, 2x and 3x of the default background that was used in the original experiments. The results are shown in figures 5a and S5a (pages 9-10, lines 206-238). We found a clear increase in AUPRC when the background signal decreases for almost all tools (with some exceptions).

In addition, we also sub-sampled data from additional ChIP-seq experiments in which antibodies against different TF and histone marks were used that resulted in different levels of background signal. We included data with different FRIP than in the initial experiments, as it is not possible to precisely control the signal-to-noise ratio with DCSsub (we cannot distinguish if a read in a peak region is signal or noise; however, the output of DCSsub is still highly dependent on the signal-to-noise ratio of the input data). The results for TF peaks confirmed the increase in AUPRCs for increased signal-to-noise ratios. However, the results for peaks reflecting different histone marks showed a different behavior. Whereas the AUPRC was higher when signal-to-noise ratios were increased for most tools in the analysis of sharp marks, the AUPRC distribution was more heterogeneous when broad marks were analyzed. In this scenario, the prediction power of most tools stayed stable, while it even dropped for some of them when signal-to-noise ratios increased. In part, this appeared to be dependent on the characteristics of the investigated histone mark. However, as the FRIP is highly dependent on sequencing depth and peak calling setup, its usefulness as a metric for the signal-to-noise ratio might be reduced for sharp and particularly for broad histone marks due to their heterogeneous peak shapes. We show these results described on pages 9-10 lines 206-238 and discussed on page 15 lines 349-357 in Figures 5b and S5b in the revised version of the manuscript.

Minor issues

Comment: The text focuses on transcription factors and hardly mentions histone modifications, yet the histone modifications are an important focus of ChIP-seq experiments, and the comparative analysis they did. The text should be modified to reflect this.

Response: We agree that a considerable part of ChIP-seq experiments is focusing on histone modifications. The perceived imbalance in our manuscript might be derived from the internal numbering scheme, which starts with the TF scenarios. Therefore, we might involuntarily mention the first test sets more often. We tried to modify the manuscript to report and discuss observations obtained from the analysis of histone marks more prominently (page 5, lines 116-117; pages 6-7, lines 142-144; page 8, lines 169-170 and lines 192-193; page 13 lines 292-296).

Comment: Its hard to tell the difference in performance in the Fig6A heatmap. Instead of alphabetical sorting, can the rows be sorted by some sort of mean/median score across the performance metrics? This will make the figure much more useful for a lab that needs to pick one or two methods

Response: We ordered the DCS tools in Figure 8a (former 6a) and Supplementary Figure S8a (former S6a) by mean DCS score over all shapes and scenarios (page 40, lines 806-807 and lines 812-813).

Comment: Is it possible to at least somewhat quantify "ease of installation"? that is also a very important consideration. for example, "order of magnitude" amount of time between having the tool downloaded and getting it up and running (e.g., minutes, hours, days...) I realize this will depend on the individual or the particular environment (and hence, sometimes luck), but it would be useful to know even roughly how hard it is to install these tools, since of course this can vary substantially from one tool to the other

Response: Although this parameter is heavily dependent on the experience of the user and the working environment, we added the column “ease of installation” in Table S2. We tried to quantify this (rather subjective) task as follows:

+++ : Execution of a few predefined lines, including all tools that can be installed via Bioconductor and pip. Installation time, seconds-minutes.

++ : Compilation/manual installation of requirements. Installation time, minutes. + : Compilation and manual installation of requirements, Installation time, minutes-

hours.

- : Manual compilation/installation of source code and requirements or modification in source code required. Installation time, hours-days.

(page 42-43, lines 866-871).

Comment: The last paragraph in the results section is long and seems to contain multiple topics - please split

Response: We included additional sub-headings in the last sections of the results (page 12, line 266 and line 279; page 14 line 315).

Comment: DCS score seems very arbitrary: "For the final score we summed up the metrics using the following weights: AUPRC 1.6, standard deviation and number of NAs 0.2, runtime 0.4, preparation time 0.2, and memory consumption 0.4." - please provide some sort of intuitive justification

Response: We added more details in the results sections to provide information on our choice of weights (page 14, lines 319-323). We also included an additional table S4 for readers interested in calculating their own scores or using different values or weights.

Comment: Lines 152-158: Highlighting the top five tool parameter setup combinations based on AUPRC values, for each regulation scenario and peak shape, showed that edgeR, DESeq2 [32] or bdgdiff/MACS2 were prominently represented among the top performing tools across all conditions (Figure 3c, top tools with exact parameter setups are shown in Additional file 4: Figure S3). MEDIPS performed very well in the analysis of sharp marks. HOMERpd [33] (peak dependent run) and DiffBind [34], both tools with previous peak calling via MACS2 or SICER2, yielded very good results for the 100: scenarios, irrespective of peak shape.

In Figure 3c, DiffBind is in the top 5 for each scenario, but the text implies the performance is good only in the 100:0 scenarios. Why is that?

Response: DiffBind (in combination with the appropriate peak caller) indeed performs very well in all scenarios. We rephrased the section accordingly (pages 7-8, lines 165- 170).

Comment: Lines 161-164: As DCS tools rank their results based on various metrics, such as p-value, FDR or diverse scores that are not compatible with a uniform threshold, we focused on the top 300 predicted regions and set the threshold just below the lowest number of differential regions present in any test case. This sentence doesn't make sense to me. What threshold?

Response: We rephrased this section to clarify that we used the top 300 regions, because at least 300 differentially regulated regions were obtained in all test cases (page 8, lines 171-175).

Comment: Lines 175-177: The FDR was below 0.2 in all cases, and even lower for histone marks, while the FOR was lower than the FDR across all samples (Figure 4b). Figure 4b shows that the FOR is not lower than the FDR for some of the broad mark samples. The text should be changed from "all samples" to "most samples"

Response: Corrected (page 8, line 189).

Comment: Lines 184-186: In this case, SICER2 derived peaks were used for the analyses with HOMERpd, DiffBind and DESeq2, MACS2 peaks for MAnorm2, and MEDIPS is peak independent.

Why was a different peak caller used for MAnorm2?

Response: All peak callers selected for this study were used in combination with MAnorm2 as well as all other peak-dependent DCS tools. In this figure, we show the accuracy profiles of the top 5 DCS tool-parameter combinations by AUPRC. For broad marks with 100:0 regulation, the top performing MAnorm2-peak caller combination was MACS2 with MAnorm2, ranking 2nd based on AUPRC. We updated the caption of Figure 4 (page 39, lines 770-771) and added a description in the respective section (page 9, lines 197-200).

Comment: Lines 231-233: The fraction of predicted regions that are shorter than the reference was between 0.002% and 0.31%. Here, the negative outliers were DBChip and MultiGPS with 0.31% and 0.29%, respectively.

I don't understand this wording - 0.31% and 0.29% aren't outliers.

Response: The sentence was rephrased (page 13, line 308).

Comment: Line 406: Duplicate single-end reads were kept. Why? That's not standard practice.

Response: Different opinions exist about handling of duplicate single-end reads. While in general they can be removed (see: Bailey T, Krajewski P, Ladunga I, Lefebvre C, Li Q, et al. (2013) Practical Guidelines for the Comprehensive Analysis of ChIP-seq Data. PLOS Computational Biology 9(11): e1003326. https://doi.org/10.1371/journal.pcbi.1003326 in Box7), we decided to keep them because we simulated and sub-sampled duplicate reads as signals. In other words, we did not obtain PCR-amplified fragments from the simulation nor from the sub-sampled data, as we wanted to make sure that the read number was kept as close as possible to the simulated/sub-sampling reference numbers. In general, the numbers of duplicated reads (identified by Picard tools) were quite low (< 0.07% of the aligned reads for all simulated/sub-sampled data sets). We rephrased this part in the methods section to make this more clear (page 23, lines 545-547).

Comment: Lines 417-422: Change the order of the sentences - describe SICER2, then MACS2

Response: Corrected (pages 23-24, lines 558-564).

Comment: Line 434: HMCan is missing the reason why it was run on the other computer.

Response: Corrected, it was also executed with 5 threads (page 24, line 577).

Comment: Lines 653-666 (Legend for Figure S4). The legends aren't associated with the correct panels. Panel "a" has the text for panel "c". Panel "b" has the text for panel "d". Panel "c" has the text for panel "a". Panel "d" has the text for panel "b". Additionally, line 659 should be "broad mark", not "sharp mark".

Response: Corrected (page 44, lines 899-906).

Comment: Include panel number when referring to supplementary figures (e.g., Figure S4a rather than just Figure S4).

Response: We added sub-panel numbers for all supplementary figures.

Comment: Provide more details on the transcription factor and histone marks (some readers may not be familiar with them). e.g., H3K27ac is found on enhancers and promoters; H3K36me3 covers gene bodies; C/EBPa is involved in ...

Response: We added information on the functions and roles of C/EBPa, H3K27ac and H3K36me3 in the results section (page 5, lines 113-114 and lines 116-117). Comment: Make sure all "Parameter setup" values in Table S3 are present in Table S2. For instance, the values for DiffBind in Table S3 don't match what is in Table S2. Response: These mismatches were caused by a different tool – parameter – peak caller combination scheme that we used internally. We updated Table S5 (former S3) so it matches Table S3 (former S2).

Comment: Provide details on uniquepeaks - there's no citation or download link in Table S1.

Response: We added a description of uniquepeaks (it is a custom approach applying BEDtools to identify uniquely bound regions, briefly described in Table S2 (former S1))

(page 6, lines 139-140).

Comment: What exactly does "off-the-shelf" mean? (Different people tend to use it slightly differently).

Response: We use the term off-the-shelf software/approach to describe standard software or an approach using software that is freely available and easily installed on almost all machines used by bioinformaticians. The two mentioned approaches use for example shell scripts, R or BEDtools. To avoid any misunderstanding we decided to use the term “custom approach” in the revised version of the manuscript (page 4, line 72; page 6, lines 126-127 and lines 139-140).

Comment: Figure 4b: The plots on the right-hand side should be the same size as the others.

Response: We re-formatted the plot in Figure 4b with equal y-axis height.

Comment: Figure S6a: The random regions aren't included (see lines 449,451)

Response: We updated Figure S3 and added the AUPRCs from the respective random regions.

Comment: Table S1: The "Focus" column is really informative. Please indicate which tools have a focus on RNA-seq.

Response: RNA-seq was added to the focus column (now Table S2).

Comment: Lines 48-50: quantitative comparison of protein-DNA interactions between different biological scenarios requires a priori knowledge of genomic regions that are occupied by the protein under investigation. Why? What types of knowledge? I don't quite follow the reasoning here. Response: We intended to say that in principle, one can either focus on the occupied regions or analyze them in a comparative fashion between samples. To do the latter, one requires the positions of the occupied regions in all samples first. We rephrased this section to make this statement more clear (page 3, lines 47-49).

Comment: Lines 56-58: For instance, some tools, particularly those initially developed for RNA-seq analysis, assume that the majority of occupied genomic regions do not differ between experimental states. Yet, this is often not the case in ChIP-seq datasets. (Regarding the statement about the ChIP-seq datasets) Really? Is this common/general knowledge or is there a citation?

Response: We rephrased this section to make our point more clear (page 3, lines 58- 62). We wanted to address ChIP-seq studies that involve experimental perturbation of levels and/or the activity of the protein under investigation. An example would be the global down-regulation of a particular histone modification or TF after treatment with small molecule inhibitors or knockdown with shRNAs. This would result in the inadequate identification of differentially occupied regions if the normalization method was based on suboptimal assumptions.

Comment: Lines 59-60: Furthermore, depending on the biological scenario, different normalization methods employed by individual tools will have a strong impact on the outcome.

Any examples/citations?

Response: We rephrased this section and added an example and citations (page 3, lines 58-62).

Typos / grammatical errors

Comment: Line 61: ranging from few hundred to several thousands

It should be "ranging from a few hundred" (missing the "a")

Response: Corrected (page 3, line 65).

Comment: Lines 74-75: In addition, our decision trees also provide recommendations for the analysis of ChIP-seq datasets of

Remove either "In addition" or "also" - including both is redundant

Response: Corrected (page 4, line 79).

Comment: Line 114: evaluation pipeline (Figure 1b), which includes, alignment against the respective reference genomes

Remove the comma after the word includes

Response: Corrected (page 6, line 119).

Comment: Lines 171-172: Furthermore, as a subset of DCS tools depends on predefined window sizes, these impacts on their predictions.

Change "predefined window sizes" to "a predefined window size"

Change "these impacts on their predictions" to "this impacts their predictions"

Response: Corrected (page 8, line 184).

Comment: Lines 224-225: In particular, RSEG was not capable to successfully deal with a global down-regulation of ChIP-seq signals in one sample.

Change "capable" to "able"

Response: Corrected (page 13, line 300).

Comment: Lines 234-236: There were no empty output files or failed execution runs for most tools, while only few runs with the MMDiff2 (14.6%) and NarrowPeaks (7.6%) tools failed.

Change "while only" to "except for a"

Change "failed" to "that failed"

Response: Corrected (page 13, line 311).

Comment: Line 236: For MMDiff2 memory allocation failed for individual peak

Remove "For" at the beginning of the sentence.

Response: Corrected (page 13, line 312).

Comment: Line 242: towards the optimal setup of DCS tool

It should be "towards the optimal setup of a DCS tool" (missing the "a")

Response: Corrected (page 14, line 324).

Comment: Line 252: we provide decision trees that will help to guide biomedical

scientists

Remove "to"

Response: Corrected (page 14, line 338).

Comment: Line 289: investigators might chose the tools which showed

Change "chose" to "choose"

Response: Corrected (page 16, line 389)

Comment: Line 293: we suggest to use the tools

Change "to use" to "using"

Response: Corrected (page 17, line 394).

Comment: Line 317: were checked against a black list

Change "black list" to "blacklist"

Response: Corrected (page 18, line 418).

Comment: Line 328: In addition, also noise was added to the samples and their

replicates.

Remove "also"

Response: Corrected (page 18, line 429).

Comment: Line 334: was stored in bed file

Change "was" to "were"

Response: Corrected (page 18, line 435).

Comment: Lines 342-343: with repeat-masked regions greater 1kb as black list.

Change to "with repeat-masked regions greater than 1kb as a blacklist."

Response: Corrected (page 19, line 445).

Comment: Line 397: Change "Hiseq2500" to "HiSeq 2500"

Response: Corrected (page 22, lines 435-436).

Comment: Line 459: and data were combined this with

Remove "this"

Response: Corrected (page 25, line 602).

Comment: Line 470: The top 300 predicted peaks

Change to "The top 300 predicted differential peaks"

Response: Corrected (page 26, line 613).

Comment: Line 473: The minimum of simulated

Change to "The minimum number of simulated"

Response: Corrected (page 26, line 616).

Comment: Line 477: The FOR was also calculated by

Remove "also"

Response: Corrected (page 26, line 621).

Comment: Line 525: used the following public available datasets

Change "public" to "publicly"

Response: Corrected (page 28, 669).

Comment: Line 625: from a H3K27ac histone mark

Change "a" to "an"

Response: Corrected (page 41, line 840).

Comment: Line 627: from a H3K36me3 histone mark

Change "a" to "an"

Response: Corrected (page 42, line 843).

Reviewer #2

The manuscript presents a comprehensive and detailed assessment of bioinformatics approaches for 'differential' analysis of ChIP-Seq data. I find the design of the pipeline to be very careful and reasonable. The conclusions are solid and practically useful and the visualization of the results is truly beautiful.

Yet, there are some important issues to be addressed.

Major

Comment: (1) As far as I understood from the Methods, the authors used a single mouse chromosome (chr19) to perform all analyses. Many top-scoring approaches were using macs2 peak calls, with macs2 being executed with 'default' settings. According to the Methods and Supplementary Data, for some reason, the genome size was not specifically set for macs2 callpeaks (see the -g parameter of macs2). It is strange, as chr19 size was explicitly specified for a competing approach JAMM (Table S2).

I am not sure how it affects the results of the peak calling in this particular setting, but it seems a major issue that could change the outcome of the downstream analysis.

Response: We aimed at using the default parameters whenever possible, as we did not intend to benchmark the performance of peak calling tools alone. While MACS2 does not require setting chromosome sizes to run, JAMM requires this information. Therefore, we executed JAMM with the chr19 chromosome size set. However, we fully agree that any potentially confounding effects associated with this step would be along in our analysis pipeline and might change the outcome for all peak-dependent DCS- tools.

To address this, we thoroughly checked the MACS2 manual and found that this tool assumes a chromosome/genome size of 2.7e9 (for Homo sapiens) if no chromosome size is specifically set using the -g parameter. Therefore, we tested the performance of MACS2 with –g 1.87e9 (mm10) and –g 58205856 (for chr19 only) and without setting –g (default value: 2.7e9). To evaluate the resulting bed files (representing peaks) we chose the bedtools Jaccard function to calculate similarity. We compared the MACS2 output bed files to the respective reference peak files from the simulation and from sub- sampling. We found minor differences between the default (2.7e9) and the mm10 (1.87e9) -g options. However, there was a slight decrease in the Jaccard similarity for chr19 only (58205856). The results are shown in Supplementary Figure S1i in the revised manuscript (page 23, lines 561-564).

We also tested the impact of the MACS2 -g parameter on the differential ChIP-seq results. To this end, we ran DiffBind with the MACS2 output files coming from simulated data representing different -g parameters and compared the AUPRCs. We found that there was almost no difference in the resulting AUPRC values between g- 1.87e9 (mm10) and the default -g parameter of 2.7e9. Furthermore, the resulting region files were almost identical. When the size of chr19 size was used for setting the –g parameter, we observed a slight increase in AUPRCs in the model mode but only for scenario/shape parameter combinations that yielded very low AUPRC values and therefore did not influence the final recommendations of our study. Hence, we decided to keep the AUPRC values as they were initially obtained, updated the methods section (page 23, lines 561-564) and added Supplementary Figure S1i to show the influence of this parameter on the peak files.

Comment: (2) It is unclear why chr19 was selected for analysis. Chromosomes are very non-uniform in terms of nucleotide composition and ChIP-Seq signal distribution, so a genome-wide estimate might provide more stable results.

As it might be unfeasible due to runtime constraints, a subset of 3-5 chromosomes could be still much more representative.

I suggest replicating at least a part of the analysis using a wider set of chromosomes (maybe re-check the performance of a small set of top-performing tools).

Response: We initially chose chr19 because of its small size, as lower length means less reads to simulate or subsample, align and process with the DCS tools. Especially for the application of the DCS tools and for the evaluation of our predictions, optimizing the time consumption of our pipeline was important, as we performed over 23,000 analyses for this study.

However, we agree that conclusions that are based on the size and nucleotide composition of one chromosome might be biased. Therefore we included chromosomes 1 (195.471.971 bp, 41% GC), 8 (129.401.213 bp, 42% GC), 11 (122.082.543 bp, 44% GC) and X (171.031.299 bp, 39% GC) in addition to chromosome 19 (61.431.566 bp, 43% GC). We simulated data for TFs, sharp and broad marks and reran the analysis for the 11 top-ranking DCS tools (we used the top five tools of each test scenario, which partially overlapped) derived from the initial AUPRC comparison. The results of the simulated data showed an interesting effect for chrX, which might be caused by its poorer quality in the mm10 assembly. These new analyses are shown in Figure 6 and S6a in the revised version of the manuscript (pages 10-11, lines 239-251).

To investigate the distribution of ChIP-seq signals we performed sub-sampling from the genuine ChIP-seq data sets using the five representative chromosomes and subsequently applied the top 11 DCS tools. Using the resulting AUPRCs we investigated if a connection between chromosome characteristic and AUPRC exists (Figure S6d). Even though we observed effects and slight trends pointing in this direction, we cannot rule out that these were limited to the chosen ChIP-seq datasets or to the proteins/histone marks that were studied (page 11, lines 252-265 and pages 15-16, lines 358-367). Although it might be interesting to investigate these effects in more detail, a thorough analysis would require the analysis of a substantial set of additional ChIP-seq data for the same and different target proteins. However, given the time constraints associated with the revision period, we believe that such an investigation would be beyond the scope of this manuscript.

Comment: (3) Only a single ChIP-Seq dataset of each 'peak shape type' was used for evaluation. It is unclear if the underlying data had low or high sequencing depth, and whether the QC metrics (e.g. those suggested by the ENCODE ChIP-Seq analysis guidelines) were fulfilled by the utilized data. This should be checked and explicitly stated in the manuscript. Similar to (2) it might be very useful to replicate at least part of the analysis on different ChIP-Seq data of the same type (TF, narrow, or broad marks), as its signal-to-noise ratio can be dramatically different depending on a cell type or antibody.

Response: We fully agree that the datasets used for our study must meet the most important quality control metrics. To address this point, we tested whether our datasets meet a number of QC metrics including those proposed by the ENCODE and ROADMAP consortia. Sequencing depth, NSC and RSC were fulfilled (according to this paper: Landt SG, Marinov GK, Kundaje A, et al. ChIP-seq guidelines and practices of the ENCODE and modENCODE consortia. Genome Res. 2012;22(9):1813-1831. doi:10.1101/gr.136184.111) for all genuine ChIP-seq data that were used for subsampling with DCSsub. We stored these metrics together with the read numbers and additional QC metrics obtained by the ssp tool in Table S1 (page 21, lines 499- 503).

To address the second point, we simulated additional test sets with higher and lower FRIP and included additional ChIP-seq data sets for TFs, sharp and broad marks. We were able to precisely adjust the signal-to-noise ratio in the simulated data with DCSsim by increasing or decreasing the background signal (0.5x, 2x and 3x of the default background used). We observed a clear increase in AUPRC for almost all tools when the background signal decreased. The results of this analysis are shown in figures 5a and S5a of the revised manuscript.

Yet, DCSsub cannot clearly distinguish between signal and noise inside the regions that were selected for the differential sub-sampling. Therefore, we used the same setting as in the initial analysis. The downside of this choice is that we had to work with the S/N ratio of the input data alone. The utilized TF (PU1 and STAT6), sharp (H3K9ac and H3K4me3) and broad mark (H3K27me3 and H3K79me2) ChIP-seq data. These had all higher or lower FRIP than our initial data sets. As expected for the simulated data, we found a clear increase of the AUPRC with decreasing background. This was true for all tested tools and for all peak shapes, with a few exceptions. Sub-sampled TF results confirmed the increase in AUPRC when the signal-to-noise ratio was increased. However, the results for the sub-sampled histone mark data showed a different pattern. While we found that for some of the tools with sharp marks the AUPRC increased when signal-to-noise ratio was going up, the AUPRC distribution was more heterogeneous when broad marks were analyzed. In part, this appeared to be dependent on the characteristics of the investigated histone mark. However, as the FRIP is highly dependent on sequencing depth and peak calling setup, its usefulness as a metric for the signal-to-noise ratio might be reduced for sharp and particularly for broad histone marks due to their heterogeneous peak shapes. We show these results on pages 9-10, lines 206-238 and in Figures 5b and S5b and discus them on page 15, lines 349-357 in the revised version of the manuscript.

Minor

Comment: Page 2 - "all physiological processes" - I would suggest rephrasing this sentence. There is plenty of cellular machinery acting w/o DNA-protein interactions, and I would avoid talking about 'physiological' processes (with ChIP-Seq, we are on the molecular level).

Response: Rephrased (page 2, line 42).

Comment: Page 3 - "qualitative or quantitative" - I understand the intention of the authors to highlight differential analysis of ChIP-Seq data, but I consider all steps of the analysis as 'quantitative'. I suggest rephrasing this sentence.

Response: Rephrased (page 3, lines 47-49).

Comment: Page 3 - "parameter-guided" what does it mean here?

Response: Rephrased (pages 3-4, lines 68-69).

Comment: Page 6 - "accuracy" - this word has a very particular technical meaning in machine learning, and I strongly suggest avoiding it when referencing a 'generic' predictive power of a model. Please consider 'performance measures', 'prediction quality' etc as possible alternatives.

Response: Rephrased (page 6, lines 129-130).

Comment: Page 7 - Here it is unclear what are "accuracy profiles" and "differentially represented" genomic regions.

Response: We rephrased this section (page 8, lines 171-175).

Comment: Page 7 - "top 300 predicted regions" - does it mean the predictions were evaluated at the fixed positive prediction rate? That is OK, but the end of the sentence is confusing "lowest number of differential regions present in any test case". What does it mean?

Response: We rephrased this section to clarify what we meant with lowest number of differential regions (page 8, lines 169-175).

Comment: Page 8 - "TF peaks were not accurately detected with edgeR" - edgeR does not perform peak calling.

Response: This part was rephrased, we used edgeR for the prediction of differential peaks (page 8, lines 180-183).

Comment: Page 8 - EpiCenter used with a narrow window for broad peaks. First, why?

Second, this is more suited for Discussion.

Response: We applied all tool–parameter combinations on all data sets, therefore also suboptimal combinations were tested. This section describes just one example of a suboptimal combination. We rephrased this section to make that more clear (page 8, lines 183-185).

Comment: Page 13 - "protein clusters". I find this terminology rather confusing. It might be better to stick to "clusters of binding sites/regions".

Response: We agree. This term stems from an earlier version of the DCSsim tool and is indeed confusing; we changed it to “clusters of binding sites” in the revised version of the manuscript (page 18, line 415).

Comment: Page 15 - "background noise was set ..." Methods lack details regarding background noise simulation.

Response: This process is now described in the methods section as follows: In addition, background noise was added to the samples and their replicates. This was done via weighted bins, which were also used to create the noise in input objects for both samples. Noise weights were sampled from a gamma distribution (page 18, lines 429-431).

Comment: Page 17 - usage of spike-ins requires a bit more details. Which particular tools were provided with external scaling factors?

Response: We added a column in Table S3 that lists the parameter-setups where we used spike-ins to derive an external scaling factor per DCS tool.

Comment: Figure 1c requires Y-axis label. Overall, the Figures clarity and style are top- notch, but figure captions are extremely brief. I would suggest providing more details.

Response: We added Y-axis labels to Figure 1c, and S1e,f,g; and provided more details in the figure captions (pages 37-47)

**2^nd^ round**

**Reviewer 1**

The authors have done an excellent job responding to all reviewer criticisms.

The additional analyses performed by the authors regarding different FRiP levels, additional chromosomes, and additional TFs and histone marks is very impressive. Their summary on lines 262-265 (copied below) is a key result that is very reassuring to those of us who perform differential analysis.

In summary, these analyses show that chromosome and ChIP-seq signal characteristics affect the AUPRC of the top-ranking tools, while we did not observe any clear advantage of specific tools for particular scenarios and/or settings.

===============================================

(Very) minor issues

Change "FRIP" to "FRiP" (lowercase i) on lines 208, 230, 232, 233, 235, 237, 238, 352, 354, 357, 468, 660, 784, 787 (twice), 790 (twice), 924, 925 (twice), 926.

Change "FRIPS" to "FRiPs" (lowercase i, lowercase s) on line 234.

Change "ROADMAP" to "Roadmap Epigenomics" on line 500.

**Reviewer 2**

I am impressed by the effort the authors put into the revised version of the manuscript.

I consider the revised version of the manuscript fully suitable for publication.

As a possible discretionary edit, I would suggest highlighting the most "universal/recurrent" (Figure 8) tools for typical usage scenarios right in the Abstract.
